# Plasma and urinary metabolomic profiles of Down syndrome correlate with alteration of mitochondrial metabolism

**DOI:** 10.1038/s41598-018-20834-y

**Published:** 2018-02-14

**Authors:** Maria Caracausi, Veronica Ghini, Chiara Locatelli, Martina Mericio, Allison Piovesan, Francesca Antonaros, Maria Chiara Pelleri, Lorenza Vitale, Rosa Anna Vacca, Federica Bedetti, Maria Chiara Mimmi, Claudio Luchinat, Paola Turano, Pierluigi Strippoli, Guido Cocchi

**Affiliations:** 1 Department of Experimental, Diagnostic and Specialty Medicine, (DIMES), Unit of Histology, Embryology and Applied Biology, University of Bologna, Via Belmeloro 8, 40126 Bologna, BO Italy; 2 CERM, Center of Magnetic Resonance, University of Florence, Via Luigi Sacconi 6, 50019 Sesto Fiorentino, Florence Italy; 3 CIRMMP, Consorzio Interuniversitario Risonanze Magnetiche Metallo Proteine, Via Luigi Sacconi 6, 50019 Sesto Fiorentino, Florence Italy; 4 Neonatology Unit, St. Orsola-Malpighi Polyclinic, Via Massarenti 9, 40138 Bologna, BO Italy; 5 Neonatology Unit, St. Orsola-Malpighi Polyclinic, Department of Medical and Surgical Sciences (DIMEC), University of Bologna, Via Massarenti 9, 40138 Bologna, BO Italy; 6 Institute of Biomembranes, Bioenergetics and Molecular Biotechnologies, National Council of Research, Via Amendola 165/A, I-70126 Bari, Italy; 7 Department of Medical and Biological Sciences, University of Udine, P.le Massimiliano Kolbe 4, 33100 Udine, Italy; 8 Department of Chemistry, University of Florence, Via della Lastruccia 3, 50019 Sesto Fiorentino, Florence Italy

## Abstract

Down syndrome (DS) is caused by the presence of a supernumerary copy of the human chromosome 21 (Hsa21) and is the most frequent genetic cause of intellectual disability (ID). Key traits of DS are the distinctive facies and cognitive impairment. We conducted for the first time an analysis of the Nuclear Magnetic Resonance (NMR)-detectable part of the metabolome in plasma and urine samples, studying 67 subjects with DS and 29 normal subjects as controls selected among DS siblings. Multivariate analysis of the NMR metabolomic profiles showed a clear discrimination (up to of 80% accuracy) between the DS and the control groups. The univariate analysis of plasma and urine revealed a significant alteration for some interesting metabolites. Remarkably, most of the altered concentrations were consistent with the 3:2 gene dosage model, suggesting effects caused by the presence of three copies of Hsa21 rather than two: DS/normal ratio in plasma was 1.23 (pyruvate), 1.47 (succinate), 1.39 (fumarate), 1.33 (lactate), 1.4 (formate). Several significantly altered metabolites are produced at the beginning or during the Krebs cycle. Accounting for sex, age and fasting state did not significantly affect the main result of both multivariate and univariate analysis.

## Introduction

Down syndrome (DS) (OMIM #190685) is the most frequent genetic cause of intellectual disability (ID), with an incidence of 1 in 691 live births^1^. The typical DS phenotype includes intellectual disability (ID), cardiovascular defects and craniofacial dysmorphisms^2^. ID, along with a typical facies (oblique eyes with epicantic folds and flat nasal bridge) and hypotonya at birth, is the most constant feature of DS and remains the main clinical problem^1,3–5^. It presents with variable severity, mainly affecting verbal skills and symbolic thought whereas relationship and affect skills are maintained^6–8^.

In 1959, Lejeune and Coll. discovered that DS is caused by the presence of full or partial chromosome 21 (Hsa21) in three copies (trisomy 21) in the cells of the affected subjects^9^. To date, valid therapeutic strategies do not exist to improve the cognitive status of persons with DS. Pelleri and Coll., studying partial (segmental) trisomy 21 (PT21), demonstrated a highly restricted “Down syndrome critical region” (HR-DSCR) of only 34 kb on distal 21q22.13 which is duplicated in all DS subjects while it is not duplicated in subjects without a diagnosis of DS^10^.

It is widely accepted that the Hsa21 gene product excess in a ratio of 3:2 when comparing trisomy 21 and normal cells is responsible for the typical features of DS^2,11–13^; however, a pathogenetic model linking specific structural and functional aspects of Hsa21 to ID in DS is not yet known.

In his studies, Lejeune hypothesized that DS could be considered a metabolic disease. In the conference talk “Vingt Ans Après”, he explained how the one carbon cycle could be involved in the pathogenesis of ID in subjects who do not have a gross anatomic defect of the brain, and he asserted: “the goal is to figure out where a link between mental deficiency and trisomy 21 should be sought”^14^.

To explain his thoughts, he compared the genotype to an orchestra in “concert”: trisomy 21 is dis-concerting^15^. That means that the chemical bases of ID in these subjects are not coordinated. Through a careful cytological and biochemical analysis, it was demonstrated that some enzymes with increased activities are encoded by genes located on Hsa21, but also by genes located on the other chromosomes. For example, superoxide dismutase 1 (SOD1) activity, which is increased by 1.5 times in trisomy 21 children, belongs to the first group, while glutathione peroxidase (GPX1), which is also increased, belongs to the second one^15^.

In this work, we performed for the first time a metabolomic analysis of plasma and urine from Down syndrome and control subjects in order to give some insight into the metabolic processes possibly changed in DS as a result of gene imbalance. Metabolomics is a fairly recent discipline focusing on comprehensive analysis of the metabolites, in a biological system^16^. It studies metabolites, small molecules, end products of the cellular processes, which are enclosed in the term “metabolome”^17^. The major challenge of metabolomics is to analyze the highest number of endogenous metabolites as possible in a more accurate way^16^. The metabolic profile could be considered an instantaneous “snapshot” of the cell physiology. Indeed, metabolomics is giving important outcomes in the clinical area, especially in identifying biomarkers or in defining disease pathophysiology^18,19^. Any of these profiles provide information that cannot be obtained directly from the genotype, gene expression profiles, or even from the proteome (the set of all the proteins expressed by the genome) of an individual^19^.

Blood serum, plasma and urine are the biological fluids generally used to examine the alterations of metabolite levels. The two main methods used to perform metabolomic analysis are: nuclear magnetic resonance (NMR) spectroscopy and mass spectrometry (MS) coupled with separation techniques^20,21^. NMR, although characterized by a lower sensitivity than MS, results to be a very appropriate platform because it is highly reproducible, quantitative, and requires minimal sample manipulation.

The aim of this work is to verify the hypothesis that specific metabolic alterations may be detected in biological fluids of subjects with DS. To model systematic alterations of metabolites in subjects with DS, we chose to analyze plasma and urine samples. Untargeted ^1^H-NMR has been used to measure the NMR-detectable part of the metabolome in these biological fluids in both subjects with DS and healthy control subjects recruited among DS normal siblings. Multivariate statistical analysis was performed to evaluate the discrimination accuracy between DS and controls on the basis of their NMR profiles. Univariate analysis was performed to identify metabolites that have significantly different concentrations in DS and control groups. All significant results have been discussed in terms of genomics and of biochemistry.

## Results

### Study Design

The main features of the analyzed cohorts and collected samples are described in the Materials and Methods section and summarized in Table 1. Given that in the pediatric age it is not always possible to collect all sample at a fasting state, to exclude the possibility that breakfast could have altered the results if patients were examined later in the morning, we performed multivariate and univariate analysis for two groups of subjects: the “all” groups include fasting and non-fasting subjects, and the “fasting” groups.

**Table 1:** Number of DS and CTRL (n° of male and female subjects of DS and CTRL).

	Plasma	Urine	Subjects	Overlap
DS	41 (M = 23, F = 18)	51 (M = 29, F = 22)	67 (M = 38, F = 29)	25 (M = 14, F = 11)
CTRL	25 (M = 12, F = 13)	20 (M = 11, F = 09)	29 (M = 14, F = 15)	16 (M = 09, F = 07)
Fasting DS	25 (M = 12, F = 13)	26 (M = 13, F = 13)	37 (M = 18, F = 19)	14 (M = 07, F = 07)
Fasting CTRL	21 (M = 09, F = 12)	14 (M = 07, F = 07)	22 (M = 10, F = 12)	13 (M = 06, F = 07)

Overlap = subjects on whom both plasma and urine withdrawals were performed. In the DS and CTRL groups there were samples from 3 siblings of 3 subjects with DS for plasma analysis and from 17 siblings of 13 subjects with DS for urine analysis.

It was not possible to obtain results for both plasma and urine from all enrolled subjects (Table 1) due to different problems at the moment of collection, sample treatment or sample analysis. This led to a reduced number of data points in comparison to the total of enrolled subjects. Nevertheless, the final sample size allowed a statistical power of 0.86 for plasma analysis and 0.83 for urine analysis (software G*Power, estimation for Wilcoxon-Mann-Whitney test, two tails, effect size d = 0.8, α = 0.05). In addition, this led to a non-specular representation of subjects with DS and their siblings in the DS and control groups, respectively, although some overlap was conserved (3 siblings of 3 DS subjects for the analysis of plasma, and 17 siblings of 13 DS subjects for the analysis of urine).

### Plasma Metabolomic Analysis

**Figure 1:**
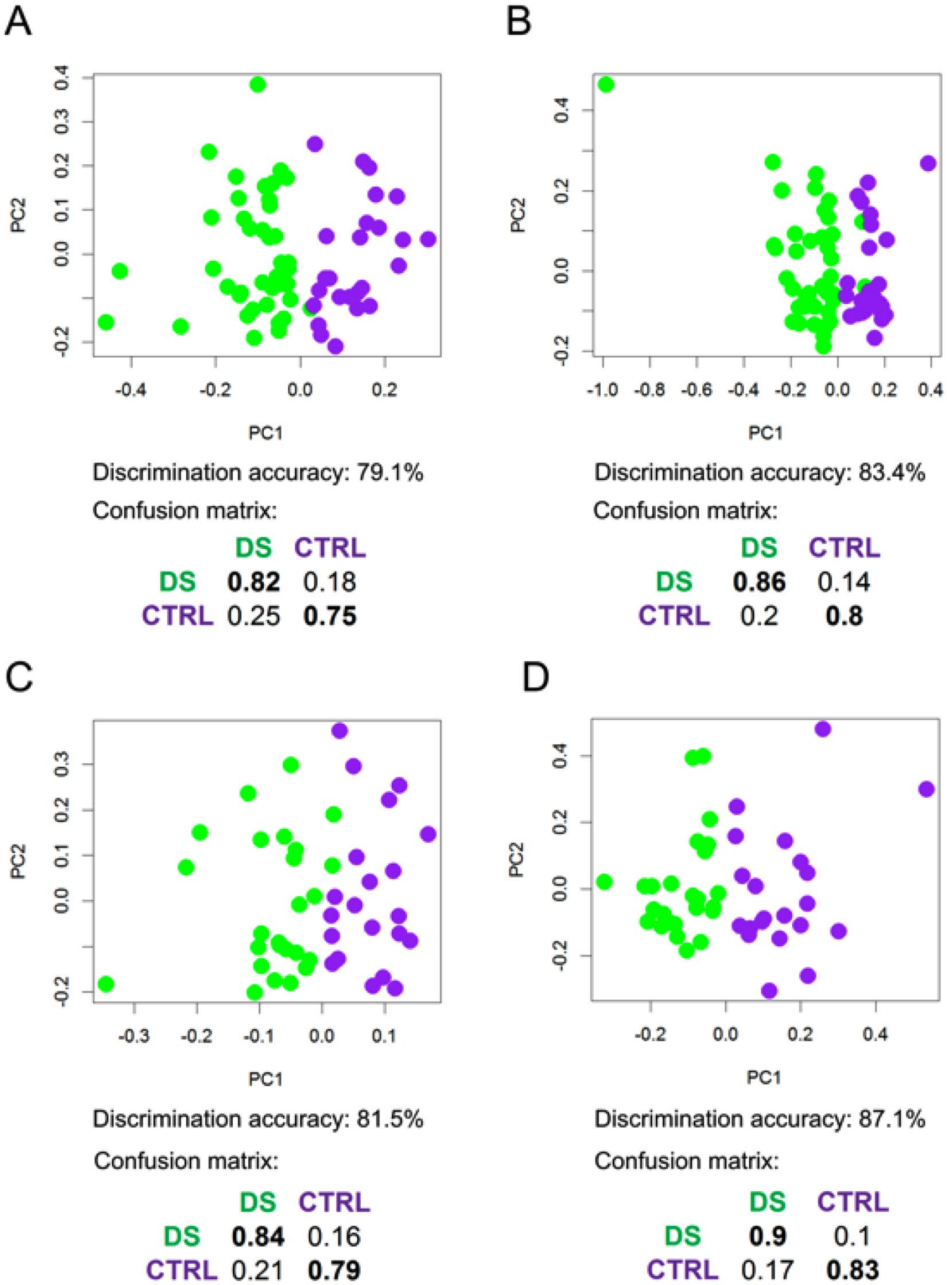
PLS-CA analysis of plasma samples. Score plots, PC1 vs PC2, and corresponding confusion matrices. In the score plot, each dot represents a different plasma sample. Green dots: DS samples; purple dots: healthy controls. All samples (DS, n = 41; CTRL, n = 25). **(A)** 1Dpresat CPMG spectra; discrimination accuracy: 79.1% (95% CI, 78.6–79.6%); sensitivity: 82.0% (95% CI, 81.3–82.4%); specificity: 75.0% (95% CI, 74.5–78.9%). **(B)** 1Dpresat NOESY spectra; discrimination accuracy: 83.4% (95% CI, 83.0–83.8%); sensitivity: 85.7% (95% CI, 85.2–86.2%); specificity: 80.0% (95% CI, 79.0–80.3%). Samples from fasting subjects (DS, n = 25; CTRL, n = 21). **(C)** 1Dpresat CPMG spectra; discrimination accuracy: 81.5% (95% CI, 80.9–82.0%); sensitivity: 83.9% (95% CI, 83.2–84.6%); specificity: 78.6% (95% CI, 77.8–79.5%). **(D)** and 1Dpresat NOESY spectra; discrimination accuracy: 87.1% (95% CI, 86.7–87.6%); sensitivity: 90.3% (95% CI, 89.8–90.9%); specificity: 83.3% (95% CI, 82.5–84.1%).

PLS-CA (Partial Least Squares-Canonical Analysis) analysis of all the plasma samples was able to discriminate DS and CTRL (Control) groups with discrimination accuracy of 79.1% (95% confidence interval (CI), 78.6–79.6%, p-value = 0.01) in 1Dpresat CPMG (Carr-Purcell-Meiboom-Gill) spectra (Fig. 1A) and of 83.4% (95% CI, 83.0–83.8%, p-value = 0.01) in 1Dpresat NOESY (Nuclear Overhauser Effect Spectroscopy) spectra (Fig. 1B). The fasting group showed discrimination accuracies of 81.5% (95% CI, 80.9–82.0%, p-value = 0.01) (Fig. 1C) and 87.1% (95% CI, 86.7–87.6%, p-value = 0.01) (Fig. 1D), respectively.

When subjects were grouped by sex, PLS-CA was able to discriminate DS and CTRL in the total female groups with an accuracy of 73.6% (95% CI, 72.9–74.3%, p-value = 0.02) in 1Dpresat CPMG spectra (Supplementary Figure 1A) and of 77.2% (95% CI, 76.5–77.9%, p-value = 0.01) in 1Dpresat NOESY spectra (Supplementary Figure 1B); in the total male groups the discrimination accuracies were 75.4% (95% CI, 74.7–76.1%, p-value = 0.01) (Supplementary Figure 1C) and 84.7% (95% CI, 84.1–85.32%, p-value = 0.01) (Supplementary Figure 1D), respectively.

A random sampling of 25 DS and 25 control subjects, repeated 50 times, still highlighted a good discrimination (80%) between DS and controls, excluding that the statistical significant difference of the mean age between the two groups may have affected the main results.

Regarding univariate analysis, the signals of 33 metabolites were unambiguously assigned (Supplementary Table S1) and integrated in ^1^H-NMR spectra of plasma. They are listed in Table 2 (all) and 3 (fasting). When all subjects were considered irrespectively of the fasting state, acetate, acetoacetate, acetone, creatine, formate, L-glutamine, glycerol, pyruvate, succinate and Unk3 (an unknown metabolite, Supplementary Figure S2) were significantly increased in DS plasma with a DS/CTRL ratio > 1; instead, lysine and tyrosine were significantly reduced in DS with a DS/CTRL ratio < 1 (Table 2). From the 1D spectral patterns, Unk3 appears to give rise to a single detectable signal at 3.94 ppm, which makes the use of 2D approaches useless for its assignment.

**Table 2:** Univariate statistical analysis of plasma samples.

Metabolites	DS (median)	CTRL (median)	DS/CTRL	p	p_FDR_	Cliff’s delta
2-Hydroxybutyrate	290.3	357.0	0.81	0.609	0.687	Negligible
3-Hydroxybutyrate	90.4	96.0	0.94	0.793	0.816	Negligible
**Acetate**	188.9	140.5	1.34*	**0**.**02**	0.063	Medium
**Acetoacetate**	77.4	44.7	1.73*	**0**.**018**	0.063	Medium
**Acetone**	92.8	72.5	1.28*	**0**.**011**	**0**.**042**	Medium
Alanine	1047.9	1071.4	0.98	0.713	0.78	Negligible
Citrate	111.7	95.4	1.17	0.086	0.167	Small
**Creatine**	146.7	78.7	1.86	**0**.**001**	**0**.**008**	Medium
Creatinine	127.9	147.0	0.87	0.101	0.178	Small
**Formate**	43.9	31.4	1.4*	**0**.**002**	**0**.**01**	Medium
**Fumarate**	2.9	2.1	1.39*	0.107	0.179	Small
Glucose	2390.5	2479.0	0.96	0.074	0.162	Small
Glutamate	93.7	77.9	1.2	0.053	0.124	Small
**Glutamine**	216.8	195.1	1.11	**0**.**008**	**0**.**036**	Medium
**Glycerol**	81.7	53.4	1.53*	**0**.**008**	**0**.**036**	Medium
Glycine	505.8	502.4	1.01	0.896	0.896	Negligible
Histidine	92.6	98.9	0.94	0.247	0.375	Small
Isoleucine	110.0	113.2	0.97	0.311	0.454	Small
**Lactate**	769.8	580.4	1.33*	0.096	0.177	Small
**Lactate + Threonine**	3912.7	2906.4	1.35*	0.05	0.124	Small
Leucine	357.3	345.9	1.03	0.528	0.661	Negligible
**Lysine**	56.2	67.7	0.83	**0**.**048**	0.124	Small
Mannose	19.9	20.2	0.98	0.609	0.687	Negligible
Methionine	203.0	177.4	1.14	0.083	0.167	Small
Phenylalanine	121.0	123.7	0.98	0.743	0.788	Negligible
**Pyruvate**	502.2	407.1	1.23	**0**.**001**	**0**.**007**	Large
**Succinate**	21.5	14.6	1.47*	**0**.**0001**	**0**.**0008**	Large
Threonine	34.9	39.1	0.89	0.183	0.291	Small
**Tyrosine**	120.4	135.7	0.89	**0**.**021**	0.063	Medium
Unk1	46.6	51.5	0.91	0.503	0.652	Negligible
Unk2	19.6	15.5	1.26	0.337	0.472	Negligible
**Unk3**	190.8	116.5	1.64*	**0**.**00001**	**0**.**0002**	Large
Valine	834.4	878.2	0.95	0.423	0.569	Negligible

List of metabolites whose concentration levels (in arbitrary units) have been determined in all samples (DS, n = 41; CTRL, n = 25). The p-value (p) of the univariate Wilcoxon-Mann-Whitney test for each metabolite is reported together with the p-value calculated after false discovery rate correction (pFDR). The effect size, using the Cliff’s delta formulation, was also calculated to aid the identification of the meaningful signals giving an estimation of the magnitude of the separation between the different groups. Metabolites that show significant concentration differences in the two groups (p-value < 0.05) and/or show values in the interval next to 3:2 are reported in bold. *Values in the interval next to 3:2 (range 1.3–1.7).

When fasting subjects were selected, the same metabolites were also found to be significantly increased or decreased in DS, with the exception of acetoacetate and lysine, which resulted not significantly different (Table 3). To evaluate the presence of confounding factors like age and sex, the metabolites which resulted significant in the univariate statistical analysis were tested with univariate and multivariate logistic regression (Supplementary Table S2).

**Table 3:** Univariate statistical analysis of plasma samples (fasting subjects subset).

Metabolites	DS (median)	CTRL (median)	DS/CTRL	p	p_FDR_	Cliff’s delta
2-Hydroxybutyrate	293.1	357.0	0.82	0.861	0.887	Negligible
3-Hydroxybutyrate	92.1	96.0	0.96	0.555	0.67	Negligible
**Acetate**	190.3	144.0	1.32*	**0**.**028**	0.109	Medium
**Acetoacetate**	71.4	53.9	1.32*	0.064	0.159	Small
**Acetone**	113.2	73.8	1.53*	**0**.**013**	0.078	Medium
Alanine	980.4	1087.0	0.9	0.219	0.349	Small
Citrate	106.5	93.3	1.14	0.09	0.203	Small
**Creatine**	127.4	78.3	1.63*	**0**.**031**	0.11	Medium
Creatinine	130.3	158.9	0.82	0.293	0.391	Small
**Formate**	41.1	31.4	1.31*	**0**.**026**	0.109	Medium
**Fumarate**	3.1	2.0	1.53*	0.098	0.203	Small
Glucose	2359.1	2471.1	0.95	0.094	0.203	Small
Glutamate	89.6	77.9	1.15	0.358	0.447	Small
**Glutamine**	208.3	195.1	1.07	**0**.**046**	0.148	Small
**Glycerol**	89.2	53.4	1.67*	**0**.**025**	0.109	Medium
Glycine	505.8	502.4	1.01	0.861	0.887	Negligible
Histidine	93.7	99.6	0.94	0.129	0.238	Small
Isoleucine	110.0	125.1	0.88	0.06	0.159	Small
**Lactate**	737.2	566.3	1.3*	0.188	0.313	Small
**Lactate + Threonine**	3850.7	2858.6	1.35*	0.141	0.246	Small
Leucine	361.3	380.5	0.95	0.273	0.391	Small
Lysine	58.9	67.7	0.87	0.113	0.22	Small
Mannose	20.0	20.7	0.97	0.662	0.742	Negligible
Methionine	198.2	177.6	1.12	0.052	0.151	Small
Phenylalanine	122.2	124.4	0.98	0.948	0.948	Negligible
**Pyruvate**	548.3	407.1	1.35*	**0**.**01**	0.072	Medium
**Succinate**	22.1	14.9	1.49*	**0**.**0001**	**0**.**001**	Large
Threonine	38.9	40.9	0.95	0.585	0.682	Negligible
**Tyrosine**	121.8	145.1	0.84	**0**.**008**	0.068	Medium
Unk1	53.4	53.7	0.99	0.678	0.742	Negligible
Unk2	20.5	14.1	1.45*	0.283	0.391	Small
**Unk3**	190.8	133.2	1.43*	**0**.**001**	**0**.**008**	Large
Valine	892.5	944.8	0.94	0.264	0.391	Small

List of metabolites whose concentration levels (in arbitrary units) have been determined in samples from fasting subjects (DS, n = 25; CTRL, n = 21). The p-value (p) of the univariate Wilcoxon-Mann-Whitney test for each metabolite is reported together with the p-value calculated after false discovery rate correction (p_FDR_). The effect size, using the Cliff’s delta formulation, was also calculated to aid the identification of the meaningful signals giving an estimation of the magnitude of the separation between the different groups. Metabolites that show significant concentration differences in the two groups (p-value < 0.05) and/or show values in the interval next to 3:2 are reported in bold. *Values in the interval next to 3:2 (range 1.3–1.7).

### Urine Metabolomic Analysis

**Figure 2:**
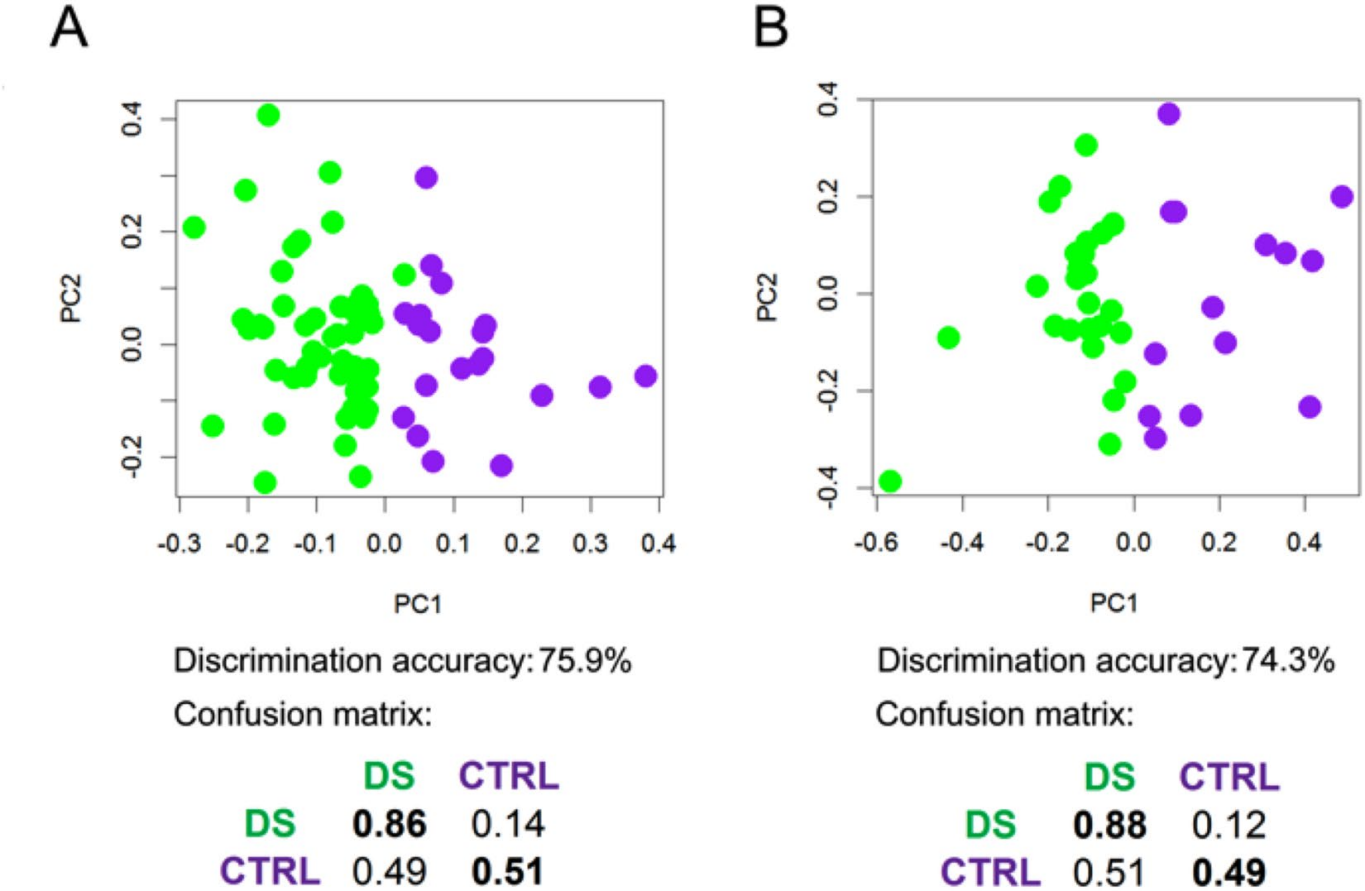
PLS-CA analysis of urine samples. 1Dpresat NOESY spectra. Score plot, PC1 vs PC2, and corresponding confusion matrices. In the score plot, each dot represents a different urine sample. Green dots: DS samples; purple dots: healthy controls. **(A)** All samples (DS, n = 51; CTRL, n = 20); discrimination accuracy: 75.9% (95% CI, 75.5–76.2%); sensitivity: 85.6% (95% CI, 85.2–86.1%); specificity: 50.9% (95% CI, 49.9–51.8%). **(B)** Samples from fasting subjects (DS, n = 26; CTRL, n = 14); discrimination accuracy: 74.3% (95% CI, 73.7–74.8%); sensitivity: 87.9% (95% CI, 87.3–88.5%); specificity: 48.9% (95% CI, 47.7–50.2%).

PLS-CA analysis of all the Probabilistic Quotient Normalization (PQN)-normalized urine spectra was able to discriminate DS and CTRL groups with an accuracy of 75.9% (95% CI, 75.5–76.2%, p-value = 0.01) (Fig. 2A). The fasting group shows a discrimination accuracy of 74.3% (95% CI, 73.7–74.8%, p-value = 0.01) (Fig. 2B).

When subjects were grouped by sex, PLS-CA was able to discriminate by 1Dpresat NOESY spectra DS and CTRL in the total female groups with an accuracy of 75.6% (95% CI, 74.9–76.2%, p-value = 0.04) (Supplementary Figure 3A) while in the total male groups the discrimination accuracy was 77.3% (95% CI, 76.6–77.9%, p-value = 0.04) (Supplementary Figure 3B).

A casual sampling of 20 DS and 20 control subjects from the total groups, repeated 50 times, still highlighted a good discrimination (71.4%) of DS vs controls, excluding that the statistical (but not clinical) significant difference of the mean age between the two groups may have affected the main results.

Regarding univariate analysis, the signals of 30 metabolites were unambiguously assigned (Supplementary Table S3) and integrated in ^1^H-NMR spectra of urine. They are listed in Table 4 (all) and 5 (fasting). When all subjects were considered irrespectively of the fasting state, phenylacetylglycine, trimethylamine-N-oxide (TMAO) and tyrosine were significantly increased in DS with a DS/CTRL ratio > 1; instead, glycine was reduced in DS with a DS/CTRL ratio < 1 (Table 4).

**Table 4:** Univariate statistical analysis of urine samples.

Metabolites	DS (median)	CTRL (median)	DS/CTRL	p	p_FDR_	Cliff’s delta
1-Methylnicotinamide	70.40	77.76	0.91	0.158	0.303	Small
2-Hydroxyisobutyricacid	475.71	466.07	1.02	0.424	0.601	Negligible
3-Hydroxyisovalericacid	580.32	664.61	0.87	0.127	0.287	Small
4-Hydroxyphenylacetate	751.67	717.87	1.05	0.47	0.639	Negligible
Acetone	183.95	151.38	1.22	0.072	0.287	Small
Alanine	619.34	764.43	0.81	0.127	0.287	Small
Allantoin	261.45	249.15	1.05	0.213	0.344	Small
Asparagine	230.67	230.47	1	0.687	0.79	Negligible
Citrate	2473.96	2223.93	1.11	0.697	0.79	Negligible
Creatine	1943.68	710.51	2.74	0.582	0.707	Negligible
Creatine + Creatinine	55388.33	66245.63	0.84	0.095	0.287	Small
Creatinine	33745.47	35848.97	0.94	0.203	0.344	Small
Dimethylamine	3221.94	3170.58	1.02	0.274	0.424	Small
Dimethylglycine	1468.8	1505.89	0.98	0.725	0.795	Negligible
Ethanolamine	611.43	557.99	1.1	0.169	0.303	Small
Formate	168.84	201.72	0.84	0.162	0.303	Small
Fumarate	23.03	24.19	0.95	0.292	0.431	Small
Glutamate + Glutamine	11171.06	10468.04	1.07	0.136	0.29	Small
**Glycine**	2376.48	3336.31	0.71**	**0**.**023**	0.153	Medium
Glycolate	1749.97	1595.02	1.1	0.764	0.812	Negligible
**Hippurate**	1759.98	2848.18	0.62**	0.123	0.287	Small
**Isoleucine**	16.84	12.89	1.31*	0.838	0.863	Negligible
Leucine	84.61	98.17	0.86	0.053	0.256	Small
Lysine	2692.58	2793.75	0.96	0.114	0.287	Small
**Phenylacetylglycine**	3291.89	2429.73	1.35*	**0**.**02**	0.153	Medium
Taurine	5402.05	5789.23	0.93	0.12	0.287	Small
**Trigonelline**	70.38	96.77	0.73**	0.109	0.287	Small
**Trimethylamine-N-Oxide**	6546.7	4502.19	1.45*	**0**.**011**	0.153	Medium
**Tyrosine**	927.51	786.09	1.18	**0**.**02**	0.153	Medium
Valine	77.15	82.48	0.94	0.913	0.913	Negligible

List of metabolites whose concentration levels (in arbitrary units) have been determined in all samples (DS, n = 51; CTRL, n = 20). The p-value (p) of the univariate Wilcoxon-Mann-Whitney test for each metabolite is reported together with the p-value calculated after false discovery rate correction (p_FDR_). The effect size, using the Cliff’s delta formulation, was also calculated to aid the identification of the meaningful signals giving an estimation of the magnitude of the separation between the different groups. Metabolites that show significant concentration differences in the two group**s** (p-value < 0.05) and/or show values in the interval next to 3:2 or 2:3 are reported in bold. *Values in the interval next to 3:2 (range 1.3–1.7). **Values in the interval next to 2:3 (range 0.58–0.76).

When fasting subjects were selected, ethanolamine, glutamate + glutamine and phenylacetylglycine were significantly increased in DS with a DS/CTRL ratio > 1; instead, leucine was significantly reduced in DS with a DS/CTRL ratio < 1 (Table 5).

**Table 5:** Univariate statistical analysis of urine samples (fasting subjects subset).

Metabolites	DS (median)	CTRL (median)	DS/CTRL	p	p_FDR_	Cliff’s delta
1-Methylnicotinamide	90.79	77.76	1.17	0.66	0.778	Negligible
2-Hydroxyisobutyricacid	534.91	466.07	1.15	0.347	0.562	Small
3-Hydroxyisovalericacid	578.97	702.61	0.82	0.21	0.385	Small
4-Hydroxyphenylacetate	776.86	717.87	1.08	0.44	0.605	Small
Acetone	177.04	157.98	1.12	0.392	0.562	Small
Alanine	641.81	716.87	0.9	0.392	0.562	Small
Allantoin	259.48	253.01	1.03	0.361	0.562	Small
Asparagine	229.66	230.47	1	0.547	0.96	Negligible
**Citrate**	2955.7	2223.93	1.33*	0.104	0.38	Small
Creatine	1623.66	710.51	2.29	0.809	0.89	Negligible
Creatine + Creatinine	55526.11	67049.14	0.83	0.146	0.385	Small
Creatinine	34711.64	40183.87	0.86	0.376	0.562	Small
Dimethylamine	3337.22	3106.79	1.07	0.2	0.385	Small
Dimethylglycine	1444.82	1402.27	1.03	0.989	0.989	Negligible
**Ethanolamine**	622.5	552.55	1.13	**0**.**045**	0.294	Medium
Formate	185.57	202.01	0.92	0.19	0.385	Small
Fumarate	22.75	23.52	0.97	0.705	0.803	Negligible
**Glutamate + Glutamine**	11376.69	10271.45	1.11	**0**.**018**	0.197	Medium
Glycine	2416.04	3117.97	0.77	0.18	0.385	Small
Glycolate	1836.13	1455.26	1.26	0.585	0.742	Negligible
Hippurate	1550.73	2898.81	0.53	0.081	0.38	Medium
**Isoleucine**	19.45	12.83	1.52*	0.64	0.778	Negligible
**Leucine**	78.61	106.32	0.74**	**0**.**003**	**0**.**042**	Large
Lysine	2643.68	2768.05	0.96	0.104	0.38	Small
**Phenylacetylglycine**	2800.31	2413.39	1.16	**0**.**029**	0.237	Medium
Taurine	5190.12	6051.34	0.86	0.154	0.385	Small
**Trigonelline**	71.63	103.65	0.69**	0.138	0.385	Small
Trimethylamine-N-Oxide	6366.2	5559.22	1.15	0.21	0.385	Small
Tyrosine	891.47	801.66	1.11	0.076	0.38	Medium
Valine	79.51	83.21	0.96	0.967	0.989	Negligible

List of metabolites whose concentration levels (in arbitrary units) have been determined in samples from fasting subjects (DS, n = 26; CTRL, n = 14). The p-value (p) of the univariate Wilcoxon-Mann-Whitney test for each metabolite is reported together with the p-value calculated after false discovery rate correction (p_FDR_). The effect size, using the Cliff’s delta formulation, was also calculated to aid the identification of the meaningful signals giving an estimation of the magnitude of the separation between the different groups. Metabolites that show significant concentration differences in the two groups (p-value < 0.05) and/or show values in the interval next to 3:2 or 2:3 are reported in bold. *Values in the interval next to 3:2 (range 1.3–1.7). **Values in the interval next to 2:3 (range 0.58–0.76).

### Genomic Analysis

We performed an analysis to correlate the metabolites we found altered in DS, the enzymes of the metabolic pathways related to these metabolites and the genomic location of the corresponding genes. The results are shown in Supplementary Table S4.

## Discussion

In this work, we have conducted for the first time a systematic analysis of the NMR-detectable part of the metabolome in subjects with DS and normal controls recruited among their siblings. Theories about DS pathogenesis, in particular concerning ID, have considered different aspects of the data derived from different research themes, focusing on neuronal proliferation, neurotransmission modulation and oxidative stress as possible main mechanisms impaired in DS. The metabolic hypothesis was presented mainly by J. Lejeune in the 70s, although related systematic investigation has not yet been performed.

To date no report has been published on the metabolomic analysis in DS, although several studies, discussed below, have underlined alterations in single compounds in blood, urine or cells obtained from DS subjects compared to normal subjects suggesting that specific alterations of metabolic pathways could play a critical role in the pathogenesis of DS^14^.

Over time several alterations on metabolite concentrations have been described in the blood of DS in comparison with control subjects: i) increased levels of phenylalanine and tyrosine in blood serum following 1-phenylalanine load and due to lower hydroxylation rate of phenylalanine^22^; ii) lower plasma levels of free histidine, lysine, tyrosine, phenylalanine, leucine, isoleucine and tryptophan^23^; iii) increased plasma concentrations of leucine, isoleucine, cysteine and phenylalanine at an age vulnerable to Alzheimer changes^24^; iv) decreased plasma concentration of serine at any age, possibly due to a dosage effect of the gene for cystathionine beta synthase (CSB), located on Hsa21^25^; v) increased plasma lysine concentration in patients above 10 years old, possibly due to premature aging^25^. More recently, concentrations of metabolites related to the methylation cycle such as cysteine, cystathionine, choline and dimethylglycine concentrations were found to be significantly elevated in DS plasma by MS analysis^26^, as well as S-adenosylhomocysteine and S-adenosylmethionine plasma level that however were found to be decreased in a previous report^27^. Discrepancies found in the results for some metabolite dosages could be due to the use of different methods or to differences in the investigated populations^26^.

Although it has been proposed that oxidative stress has a main role in the pathogenesis of DS, urinary biomarkers of oxidative stress have not been studied in this condition. A urine tyrosyl radical produced from the oxidation of L-tyrosine by the myeloperoxidase-H_2_O_2_ system of macrophages and neutrophilis^28^, has been proposed as an oxidative stress biomarker in hypothyroid DS children^29^.

Metabolomic studies were also conducted on amniotic fluid samples from fetuses with DS compared with those of non-syndromic fetuses by MS analysis showing an elevation of phenylpyruvate that inhibits the metabolism of tetrahydrobiopterin^30^; decreased levels of glycine and glutamate, involved in the neurotransmission processes^31^, and an increased level of glutamine were also measured by high-performance liquid chromatography (HPLC).

The recent availability of powerful techniques opens the way to investigate a large number of metabolites in biological fluids. This allows the measurement of the concentration of specific metabolites of interest by analyzing the spectra that are acquired for the whole complement of substances present in the fluid. On the other hand, using plasma and urine as the study models provides the possibility to visualize a balance of the metabolism at the level of the whole body. This makes sense in a genetic condition in which all cell types in all organs present with the same basic defect.

A first key result is the multivariate analysis of the data obtained from the plasma analysis, which has allowed us to discriminate with accuracies of the order of 80% changes in the NMR metabolomic profile between the DS and normal groups (Fig. 1).

The univariate analysis was based on 33 metabolites, whose NMR signals were identified and integrated in plasma spectra (Supplementary Table S1 and Tables 2 and 3). The results showed a systematic deviation for a subgroup of the analyzed substances, including several metabolites involved in central metabolic processes related to mitochondrial metabolism such as the Krebs cycle, glycolysis and oxidative phosphorylation (OXPHOS) in DS.

Metabolomic analysis by NMR provides highly precise measures of the relative concentration of the metabolites possibly affected by the fasting state of the subject when collecting the investigated fluid. Remarkably, univariate analysis revealed, even correcting for fasting state, a high level of specific change of metabolite in DS samples. Sex of the subjects was uniformly distributed in our groups (Table 1). Actually, also accounting for sex did not significantly affect the main result that plasma metabolome profile is different in DS vs normal subjects (Supplementary Figure S1). Regarding age, it is well-known that the risk of having a child with DS increase with the maternal age^32^. Therefore, children with DS are often younger than their siblings, constituting the control group. This led to a statistically significant difference between the mean age of the DS group vs control group (DS = 11.8 ± 7.1 and control = 16.8 ± 7.6, mean age ± SD). However, we have considered the critical advantage of having normal siblings of DS subjects as controls in the study of a genetic disease as the most similar genetic background is obtained between the two groups, suggesting that differences may have been due to the extra chromosome in the affected siblings rather than a more generalized genetic difference. In addition, the casual multiple resampling of subgroups with different mean ages fully confirmed the difference between the two groups. Moreover, the significant metabolites identified by univariate statistical analysis (Tables 2 and 3) maintained their significance when adjusted for sex and age factors using a multivariate logistic regression model (Supplementary Table S2). Previous works have demonstrated that dosable enzymes whose genes are located on Hsa21 adhere to the 3:2 overexpression model expected in trisomy 21^33–36^. Therefore, we have chosen to consider a metabolite to be increased based on either a statistically significant difference or a biologically significant ratio of the DS/normal median near to 3:2 (1.5), although not statistically significant due to the distribution of the values. An analogous reasoning was applied to identify a decreased concentration. Interestingly, there was a basic scheme with two alternatives: a DS/normal ratio very near to 1, implying that the metabolite is not involved in an alteration of metabolism in DS, or a ratio very near to 3:2 or 2:3, consistent with the hypothesis that the pertinent kinetic reactions are actually proceeding at 150% of their normal velocity, with the consequent yield of 150% or 67% of the relative final product, depending on the structure of the pathway. It should be noted that several significantly increased metabolites with 3:2 ratio with respect to healthy control samples are metabolic products of enzymes whose genes, except for one, are not located on Hsa21 (see Supplementary Table S4) suggesting that the interactions of Hsa21 gene products with other genes or proteins are potentially responsible for DS phenotypic variations. Interestingly, the metabolites we found increased are pyruvate, which connects Krebs cycle and glycolysis; fumarate and succinate intermediates of Krebs cycle; lactate, the end product of anaerobic glycolysis; formate, involved in mitochondrial one-carbon metabolism; and creatine, involved in the process of energy-dependent muscle activity. It is important to underline that the increment of pyruvate, succinate, formate and creatine are significant in terms of p_FDR_ and classified as medium or large in terms of Cliff’s delta (Tables 2 and 3). These alterations suggest a systematic imbalance of the Krebs cycle and different pathways of mitochondrial metabolism. Although several works have pointed out the role of mitochondria in DS^37,38^, no description of an alteration of the metabolites related to the Krebs cycle had been reported to date in DS. Krebs cycle is a central metabolic pathway for regulation of cell metabolism and energy homeostasis^39^. Accumulation of intermediate metabolites of Krebs cycle could suggest a systematic imbalance of this cycle in DS. Consistent with our results, impairment of certain enzymes of Krebs cycle (i.e. aconitase and NADP-linked isocitrate dehydrogenase) have been reported in the heart of DS fetuses and brain regions of subjects with DS^40,41^.

The increase of plasma lactate we found in DS samples is consistent with the increase of basal levels of lactate found in fibroblasts from DS patients^42^ and supports the hypothesis that in DS cells, in which the OXPHOS is impaired^42,43^, DS cells activate glycolysis for their energy demands. Consistently, also in another neurodevelopmental disease such as the autism spectrum disorder associated with mitochondrial metabolism impairment^44^, abnormal levels of metabolites associated with activation of glycolysis like serum lactate and pyruvate were found^45^. Very interesting and new data is the increase of plasma creatine in DS samples. Creatine is phosphorylated in mitochondria by ATP derived from oxidative phosphorylation and the phosphocreatine, subsequently exported outside mitochondria, used by the cytosolic creatine kinase to resupply ATP for muscle activity^46^. We can suppose that plasma accumulation of creatine, probably due to the OXPHOS impairment, could account for the muscle weakness, another typical DS phenotype.

Defective mitochondrial biogenesis has also been extensively described in DS^37^. Indeed, the overexpression of the Hsa21 gene *NRIP1* (21q11.2–21q21.1) is known to impair the activity of the transcriptional coactivator PPARGC1A causing mitochondrial dysfunction reverted by the PPARGC1A expression inducer drug metformin^47^. Impairment of the methyl cycle has been actually documented and also affects mitochondrial methyl availability and glutathione levels in DS^48^. Finally, a work of Coppus and Coll.^49^ has confirmed some metabolic alterations in DS. By HLPC, they found, for example, a decrease of tyrosine in DS compared with controls. Here, we also observed decreased tyrosine levels (not significant in terms of p_FDR_ but classified as medium in terms of Cliff’s delta (Tables 2 and 3)) with a ratio DS/control = 0.87, which was near the 2:3 ratio. Tyrosine is a precursor of thyroid hormones, whose level is often decreased in DS, so it would be interesting to test the hypothesis of a correlation between tyrosine and thyroid hormones in subjects with DS. Our preliminary analysis failed to find such a correlation when routine laboratory analysis data for thyroid hormone levels in the children investigated here were correlated to plasma tyrosine levels.

**Figure 3:**
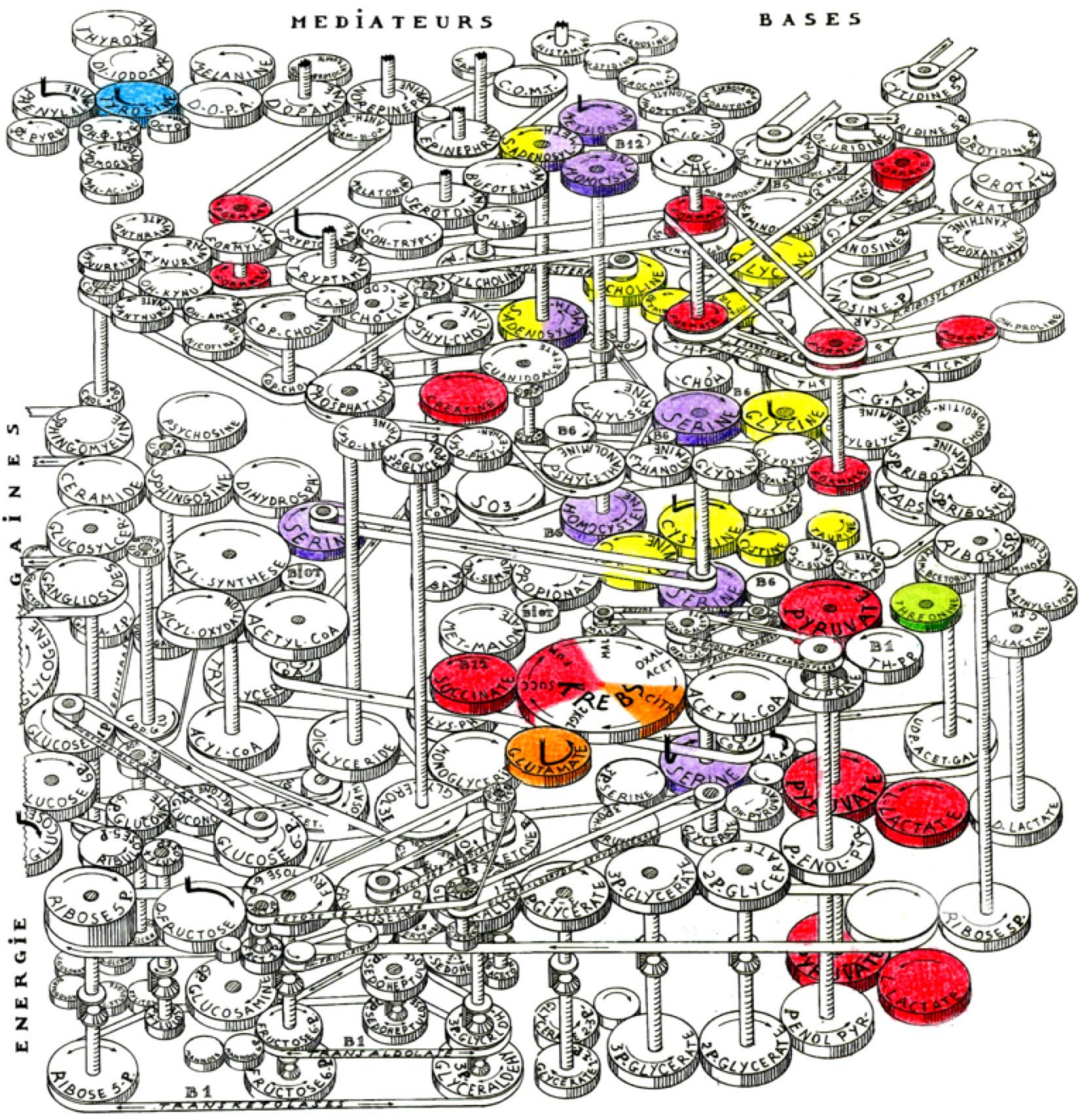
The “Lejeune Machine”, representing human metabolism as a mechanical machine, with highlighting of metabolites (gears) with an altered concentration in DS plasma. The original Figure is a drawing by Jérôme Lejeune and it has been obtained thanks to courtesy of Mme Birthe Lejeune and used with her kind permission. The drawing has been modified here by the authors by means of coloring. Red = increased (at p < 0.05 and/or with a 1.3–1.7 DS/CTRL ratio); orange = increased, although at p ≥ 0.05 and not within the 1.3–1.7 ratio range; blue = decreased (at p < 0.05 and/or with a 0.58–0.76 DS/ CTRL ratio); green = decreased, although at p ≥ 0.05 and not within the 0.58–0.76 ratio range; yellow/violet = increased/decreased, according to literature data, respectively^24–27,49^. S-adenosylhomocysteine and S-adenosylmethionine plasma level were decreased according to Pogribna and Coll.^27^, and increased according to Obeid and Coll.^26^. The yellow gear above the “Krebs” gear represents cystathionine. Other explanations in the text.

As far as the possible relationship between metabolomic profile in DS and ID are concerned, we used the “Lejeune machine”^14^ to identify which biochemical pathways presumably involved in ID were interested, according to our and previously published data for plasma (Fig. 3).

The four sections of the machine, decades before current system biology diagrams, represent the critical conditions for the nervous system to work: neuron proliferation (DNA “bases”), neurotransmission (“Mediateurs”), nerve fiber insulation (“Gaines”) and availability of energy (“Energie”). According to this scheme, our data suggest that there is a key alteration in the production of energy. To cite J. Lejeune: “Even if the network is correct and the insulating system properly developed, genetic mistakes can prevent the function. Generally speaking, one gets the feeling that the machine is running but cannot develop its full power. Exactly like a motor to which the fuel is not provided in correct amount”; “One would believe that either the brain does not dispose of enough energy or that some toxic is impairing its ignition process”^14^.

At variance with blood, urine is a biofluid characterized by a large daily variability due to the effect of food/beverage intake and a possible modulation of the urine metabolome by the circadian clock^50^. Dilution resulting by different hydration status of the donors was corrected by using PQN normalization. Nevertheless, usually, multiple collections of urine samples from the same individual are needed to extract the characteristic individual phenotype from the urinary “metabolic” noise^50–54^. Here, it was possible to obtain a single urine sample per donor, still, some meaningful differences were detectable between control and DS subjects, which complement the results obtained on plasma. The discrimination between DS and control groups by multivariate analysis appeared essentially unaffected by fasting state, sex or age (Fig. 2 and Supplementary Figure S3). This result is of particular interest because of the above-discussed fluctuation of metabolite concentrations in urine. A lower number of metabolites was found to be altered by univariate analysis (none significant in terms of p_FDR_, except for leucine in fasting subjects) still confirming that metabolites implied in key steps of the general metabolism are altered (Tables 4 and 5).

Higher levels of citrate have been previously reported in peripheral blood mononuclear cells and lymphoblastoid cells from children with DS^55^. Here, we found an increase of citrate in urine of fasting subjects with a ratio DS/control = 1.33, which was near the 3:2 ratio, although the differences in citrate levels between the two groups are not significant.

To verify if enzymes involved in pathways related to the metabolites that we have found to be significantly altered in DS are located on Hsa21, we have mapped the genomic location of these enzymes (Supplementary Table S4). The currently analyzed set revealed only one enzyme gene, *FTCD*, located on 21q22.3 and encoding for the formimidoyltransferase cyclodeaminase. FTCD enzyme is involved in the most common inborn error of folate metabolism due to an autosomal recessive disorder causing a glutamate formiminotransferase deficiency^56^. This enzyme catalyzes two reactions of the histidine metabolism, in particular the degradation of N-formimino-L-glutamic acid to form 5,10-methenyltetrahydrofolate, L-glutamate, and ammonia (KEGG pathway 2.1.2.5, Supplementary Table S4). L-glutamate is one of the compounds having an increased level in DS urine of the fasting group subjects, not significant in terms of p_FDR_ but classified as medium in terms of Cliff’s delta (Table 5). Further study is needed in this regard by analyzing other metabolites. A possible explanation of mapping only one Hsa21 gene among those found by KEGG pathway database and listed in Supplementary Table S4 could be that the alteration of an enzyme or regulatory gene on Hsa21 propagates its 3:2 effect on subsequent steps of the metabolic chain, thus affecting enzymes located on other chromosomes. From this point of view, a deep analysis of the recently described HR-DSCR, suspected to contain unknown fundamental genetic determinants for ID in DS^10^, is recommended. Although the size of HR-DSCR is lower than the mean size of a single protein-coding gene^57^, this mean size is mainly due to introns whose size may also be extremely low^58^, and there is also the possibility that short non-coding or micro-RNA might be encoded in this currently “desertic” region. The deletion of a single copy of HR-DSCR from trisomic cultured cells via CRISPR/Cas9^59^ could also allow the demonstration of phenotypic effects, and the isolation of effects on metabolism in DS due to HR-DSCR from the ones due to Hsa21 genes distant from this critical region.

Another interesting metabolite that, although not significant in terms of p_FDR_ but classified as medium in terms of Cliff’s delta, increases in urine is TMAO. This metabolite is not processed by enzymes produced by human genes but it has been hypothesized to play a role of the human microbiota. A literature analysis confirmed that TMAO is a gut-microbiota-dependent metabolite^60^, and several works also reveal that TMAO has a role in the onset of cardiovascular diseases^61–63^ and kidney diseases^64–66^. Biagi and Coll.^67^ conducted a study that analyzed the gut microbiota (GM) in DS subjects, considering the premature aging that occurs in the DS may be due to changes in GM. Deterioration of GM plays an important role in the aging of the general population as well^68^. This study revealed that DS GM is predominantly composed of Firmicutes, Actinobacteria and Bacteroidetes. The most represented families in DS GM were Ruminococcaceae (39%) and Clostridiales (9%)^67^. These bacteria are positively associated with TMAO levels^60^, so further analysis could be conducted to understand its association with DS.

Our study reveals that DS subjects present some alterations in metabolic pathways; however, more analyses are necessary to find out what is the main mechanism that determines this unbalanced concentration of some metabolites. The NMR approach used resulted extremely powerful in providing an efficient high-throughput untargeted picture of the metabolic fingerprint of the DS subjects. Nevertheless, the technique suffers sensitivity limitations. Only metabolites with concentrations ≥1 μM are measurable with confidence. Obtaining a confirmation of the proposed alteration in metabolic pathways would require targeted mass spectrometry analyses, which are beyond the scope of the present study. Possible evolutions might also be the determination of absolute rather than relative concentration of metabolites, the incorporation of a larger body of metabolome data in a metabolic network model^69,70^ and the study of the relationship between each metabolite and the protein or mRNA expression level of enzymes involved in its processing, e.g. generating quantitative, validated transcriptome maps providing DS/normal tissue ratios such as those already available for normal human tissues^71^. In addition, while in this work we focused on the diagnosis of DS as the invariant phenotype to be studied at the biochemical level, further characterization of variability within our cohort of subjects at both the genetic level (e.g., single nucleotide polymorphisms - SNP - analysis) or phenotypic level (in particular, quantitative assessment of the grade of ID) could uncover fine relationships between DS features able to explain part of the variability observed in DS, for instance a more severe grade of disease in presence of a more clear deviation of the concentration of a specific metabolite.

Thinking of DS as a metabolic disease would result in a change of perspective, especially from the point of view of possible treatment. The focus must be shifted from what is upstream (gene excess or gene defect) to what is downstream (gene product). The “blocked” mechanism that determines ID severity and specific molecule protagonists of this complex mechanism might be identified, as occurred for other complex diseases: “Phenylketonuria, galactosemia, vitamine B6 dependant homocystinuria, to take few examples, can be properly handled and the children protected against mental deficiency. Who could believe that during the coming years no new progress will be achieved?”^14^.

## Materials and Methods

### Ethics Statement

The study was approved by the independent Ethics Committee of the University Hospital St. Orsola-Malpighi Polyclinic, Bologna, Italy. Informed written consent was obtained from all participants. It was required for the patient, if over 18, or parents, to sign the informed consent for the collection of urine, blood and clinical data to participate in the study. All methods were performed in accordance with the Ethical Principles for Medical Research Involving Human Subjects of the Helsinki Declaration.

### Case selection

A total of 137 children/young adults were recruited to the study from February 3, 2014 to December 12, 2016, including 97 with DS and 40 healthy children/young adults that were siblings of the children with DS but with no evidence of abnormal karyotypes. Inclusion criteria for children with DS were diagnosis of Down syndrome with homogeneous or mosaic trisomy 21 and age > 2 years. Exclusion criteria for children with DS were distress at birth, severe prematurity (gestational age < 35 weeks) or severe neurologic disease at birth. The study has been proposed to all subjects consecutively admitted to the Day Hospital of the Neonatology Unit, Sant’Orsola-Malpighi Polyclinic, Bologna, in the context of the routine follow up provided for DS and matching the above-mentioned criteria.

Regarding the metabolomic analysis planned in the study, we were able to perform analysis and obtain results only in a subset of this group (Table 1) due to the following reasons: impossibility of obtaining an urine sample during the visit; impossibility of obtaining an adequate volume of blood during the collection; uncertainty about the fasting state of the subject; a delayed treatment of the sample following its transfer from the Day Hospital to the University Laboratory (>2 hours); a macroscopic alteration of the blood/urine sample following centrifugation. Fasting before biological sample withdrawal was preferred, however if the patient had had breakfast before blood and/or urine collection, drinks and food assumed after midnight were recorded.

The metabolomic results were eventually obtained from a total of 67 children with DS (mean age = 11.3 yrs, ± 7.0 Standard Deviation - SD) and 29 control subjects (CTRL, mean age = 16.6 yrs ± 7.8 SD). The sex distribution of both DS and CTRL was similar (Table 1), as confirmed by lack of significant differences by Fisher’s test.

### Plasma and Urine Sample Preparation

Preanalytical treatment of blood and urine samples followed standard operating procedures^72,73^. All procedures were conducted carefully and in sterility to avoid contaminations.

Blood samples were collected in EDTA-coated blood collection tubes and kept at room temperature. They were treated within two hours of blood draw. The sample was transferred to a new tube and centrifuged at 1250 g for 10 min. The plasma fraction was isolated and centrifuged for a second time at 800 g for 30 min and the supernatant was transferred to new tubes without touching the pellet or the bottom of the tube and divided in aliquots of 300 µL. All plasma samples were rapidly stored in a −80 °C freezer and ready for subsequent analysis. The exclusion criteria of plasma samples from the subsequent analysis were blood sample treatment after two hours from the draw and evident contamination of plasma samples by residual erythrocytes at the end of the treatment.

Urine samples were collected in a sterile plastic cup with lid and kept refrigerated at + 4 °C if immediate processing was not possible. They were treated within two hours of collection. The sample was transferred to a new tube and centrifuged at 2500 g for 5 min at + 4 °C (refrigerated centrifuge). After centrifugation, filtration by 0.20 µm cut-off filter was performed in order to avoid contamination of the metabolome with soluble molecules derived from cellular components. The filtered urine was transferred to sterile cryovials making 1.0 ml aliquots. All urine samples were rapidly stored in liquid nitrogen and ready for subsequent analysis.

The exclusion criteria of urine samples from the subsequent analysis were urine sample treatment two hours after the collection and formation of sandy sediment after centrifugation.

### NMR sample preparation

NMR samples were prepared according to standard procedures^72^.

Frozen samples were thawed at room temperature and shaken before use.

A total of 300 µL of each plasma sample was added to 300 µL of a phosphate sodium buffer (70 mM Na_2_HPO_4_; 20% (v/v) ^2^H_2_O; 0.025% (v/v) NaN_3_; 0.8% (w/v) sodium trimethylsilyl [2,2,3,3–^2^H_4_]propionate (TSP) pH 7.4); a total of 750 μL of each urine sample was centrifuged at 14000 g for 5 min, and 630 μL of the supernatant was added to 70 μL of a potassium phosphate buffer (1.5 M K_2_HPO_4_, 100% (v/v) ^2^H_2_O, 10 mM sodium trimethylsilyl [2,2,3,3−^2^H_4_]propionate (TMSP) pH 7.4).

The mixtures were homogenized by vortexing for 30 s and a total of 600 μL of each mixture was transferred into a 5.00 mm NMR tube (Bruker BioSpin, Rheinstetten, Germany) for analysis.

### NMR experiments

The NMR analysis has been conducted at CERM, the Magnetic Resonance Center of the University of Florence, Sesto Fiorentino (FI), Italy. ^1^H-NMR spectra for all samples were acquired using a Bruker 600 MHz spectrometer (Bruker BioSpin) operating at 600.13 MHz proton Larmor frequency and equipped with a 5 mm PATXI ^1^H-^13^C-^15^N and ^2^H-decoupling probe including a z axis gradient coil, an automatic tuning-matching (ATM) and an automatic and refrigerate sample changer (SampleJet, Bruker BioSpin). A BTO 2000 thermocouple served for temperature stabilization at the level of approximately 0.1 K at the sample. Before measurement, samples were kept for 5 minutes inside the NMR probe head, for temperature equilibration at 300 K or 310 K in the case of urine or plasma samples, respectively.

Plasma is a heterogeneous mixture composed of thousands of metabolites as well as macromolecules like proteins and lipoproteins. Due to its intrinsic characteristic, for each plasma sample, three monodimensional ^1^H NMR spectra were acquired with water peak suppression and different pulse sequences that allowed the selective observation of different molecular components: (i) a standard NOESY (Nuclear Overhauser Effect Spectroscopy)^74^ 1Dpresat (noesygppr1d.comp; Bruker BioSpin) pulse sequence, using 32 scans, 98,304 data points, a spectral width of 18,028 Hz, an acquisition time of 2.7 s, a relaxation delay of 4 s and a mixing time of 0.1 s. This pulse sequence is designed to obtain a spectrum in which both signals of metabolites and high molecular weight molecules (lipids and lipoproteins) are visible. (ii) a standard CPMG^75^ (cpmgpr1d.comp; Bruker BioSpin) pulse sequence, using 32 scans, 73,728 data points, a spectral width of 12,019 Hz and a relaxation delay of 4 s. This pulse sequence is designed for the selective observation of small molecule components in solutions containing macromolecules. (iii) a standard diffusion-edited^76^ (ledbgppr2s1d.comp; Bruker BioSpin) pulse sequence, using 32 scans, 98,304 data points, a spectral width of 18,028 Hz and a relaxation delay of 4 s. This pulse sequence is designed for the selective observation of macromolecule components in solutions containing small molecules; the resulting spectrum is generally made up only of the lipid, lipoprotein and protein signals.

Urine is a very complex biofluid but, unlike plasma, it is mainly composed of low molecular weight metabolites. Thus, for each urine sample, only a monodimensional ^1^H NMR spectra was acquired with water peak suppression and a standard NOESY^74^ pulse sequence using 32 scans, 98304 data points, a spectral width of 18028 Hz, an acquisition time of 2.7 s, a relaxation delay of 4 s and a mixing time of 0.1 s.

Acquisition of plasma and urine samples lasted approximately 4 days of NMR time. Samples of DS subjects and controls were mixed and acquired in a totally random order to avoid any batch effects.

### NMR spectral processing and analysis

Free induction decays were multiplied by an exponential function equivalent to a 0.3 Hz line-broadening factor before applying Fourier transform. Transformed spectra were automatically corrected for phase and baseline distortions and calibrated. All the urine and plasma spectra were calibrated to the reference signal of TMSP at δ 0.00 ppm, and to the glucose doubled at δ 5.24 ppm, respectively, using TopSpin 3.5 (Bruker BioSpin).

Each spectrum in the region 10.00–0.2 ppm was segmented into 0.02 ppm chemical shift bins, and the corresponding spectral areas were integrated using the AMIX software (Bruker BioSpin). Binning is a means to reduce the number of total variables and to compensate for small shift in the signals, making the analyses more robust and reproducible.

For urine samples, normalization was applied on the obtained bins to minimize dilution effects caused, for example, by variation in fluid intake; the area of each bin was normalized using PQN, calculated with exclusion of the water region (4.40–5.00 ppm).

Unlike urine, blood-plasma is not affected by dilution effects and solute concentrations are tightly controlled; thus plasma spectra do not require normalization. Plasma spectra were directly analyzed by excluding the bins containing both EDTA signals (regions: 2.53–2.60, 2.68–2.73, 3.07–3.24, 3.58–3.64 ppm) and water (region: 4.40–5.00 ppm).

No scaling of the binned data was performed; the data are only mean-centered before multivariate statistical analyses.

### Statistical analysis

Various kinds of multivariate statistical techniques were applied on the obtained bins using R 3.0.2 in house scripts.

Unsupervised Principal Component Analysis (PCA) was used to obtain a preliminary outlook of the data (visualization in a reduced space, cluster detection, screening for outliers, presence of batch effects or instrumental bias).

Partial Least Squares (PLS) was employed to perform supervised data reduction and classification between samples from healthy and diseased volunteers. Canonical Analysis (CA) was used in combination with PLS to increase supervised data reduction and classification.

The global accuracy for classification was assessed by means of a Monte Carlo validation scheme. Accordingly, each dataset was randomly divided into a training set (90% of the data) and a test set (10% of the data). The training set was used to build the model, whereas the test set was used to validate its discriminant and predictive power; this operation was repeated 200 times. For each model, the resultant confusion matrix was reported and its discrimination accuracy, specificity and sensitivity were estimated according to standard definitions. Their confidence intervals (95%) are provided in the Figures’ legends. Each classification model was also validated using permutation test; the permutation was repeated 100 times and the resulting p-value was calculated.

The metabolites, whose peaks in the spectra were well defined and resolved, were assigned (Supplementary Tables S1 and S3) and their levels analyzed. The assignment procedure was made up using an internal NMR spectral library of pure organic compounds, public databases such as the Human Metabolome Database^77^, stored reference NMR spectra of metabolites, spiking NMR experiments and using literature data^78,79^. Matching between new NMR data and databases was performed using the AMIX software.

Before univariate analysis, each metabolite was aligned to a reference value of chemical shift, obtaining a perfect alignment among all the spectra. The relative concentrations of the various metabolites were calculated by integrating the corresponding signals in defined spectral range^80^, using a home-made tool for signal deconvolution. Not assigned signals were labeled as unknown.

The nonparametric Wilcoxon-Mann-Whitney test was used for the determination of the meaningful metabolites; a p-value < 0.05 was considered statistically significant. In order to reduce false discoveries, False Discovery Rate correction (FDR) was then applied using the Benjamini and Hochberg method^81^ and the resulting p-values are reported as p_FDR_.

The effect size, using the Cliff’s delta (Cd) formulation^82^, was also calculated to aid in the identification of the meaningful signals giving an estimation of the magnitude of the separation between the different groups. The magnitude is assessed using the thresholds provided in Romano and Coll.^83^, i.e. |Cd| < 0.147 “negligible”, |Cd| < 0.33 “small”, |Cd| < 0.474 “medium”, otherwise “large”.

Univariate and multivariate logistic regression models were applied to the most significant metabolites to assess the presence of confounding factors such as sex and age of the children and the respective ODD ratios and p-values were calculated.

### Genomic Analysis

The KEGG pathway database (http://www.genome.jp/kegg/pathway.html) has been used to identify key enzymes upstream or downstream of the metabolite with an altered concentration in DS subjects. NCBI Gene database (https://www.ncbi.nlm.nih.gov/gene) has been used to search for the chromosomal location of these enzymes in order to verify a specific role of Hsa21.

### Literature Analysis

The Human Metabolome Database (http://www.hmdb.ca/) and PubMed database (https://www.ncbi.nlm.nih.gov/pubmed/) have been used to search for previous descriptions in human plasma and urine of the metabolites with an altered concentration in DS.

### Data availability

The datasets generated and analyzed during the current study are made available as Supplementary Datasets S1 (plasma data) and S2 (urine data).

## Electronic supplementary material

Dataset 1

Dataset 2

Supplementary Figures and Tables
