# Supplementary material for: Plasma and urinary metabolomic profiles of Down syndrome correlate with alteration of mitochondrial metabolism
Source: Sci Rep. 2018 Feb 14;8:2977. doi: 10.1038/s41598-018-20834-y (PMC5813015; doi:10.1038/s41598-018-20834-y)
Supplement: Supplementary file 3 — Supplementary Figures and Tables [file 41598_2018_20834_MOESM3_ESM.pdf]

Supplementary Material

Manuscript Title

Plasma and urinary metabolomic profiles of Down syndrome correlate with alteration of mitochondrial metabolism

Authors

Maria Caracausi[1†], Veronica Ghini[2,3†], Chiara Locatelli[4], Martina Mericio[5], Allison Piovesan[1], Francesca Antonaros[1], Maria Chiara Pelleri[1], Lorenza Vitale[1], Rosa Anna Vacca[6], Federica Bedetti[5], Maria Chiara Mimmi[7], Claudio Luchinat[2,8], Paola Turano[2,8], Pierluigi Strippoli[1]* and Guido Cocchi[5]

† These Authors equally contributed to the work

Affiliations

[1]Department of Experimental, Diagnostic and Specialty Medicine, (DIMES), Unit of Histology, Embryology and Applied Biology, University of Bologna, Via Belmeloro 8, 40126 Bologna, BO, Italy.

[2]CERM, Center of Magnetic Resonance, University of Florence, Via Luigi Sacconi 6, 50019 Sesto Fiorentino, Florence, Italy.

[3]CIRMMP, Consorzio Interuniversitario Risonanze Magnetiche Metallo Proteine, Via Luigi Sacconi 6, 50019 Sesto Fiorentino, Florence, Italy.

[4]Neonatology Unit, St. Orsola-Malpighi Polyclinic, Via Massarenti 9, 40138 Bologna, BO, Italy.

[5]Neonatology Unit, St. Orsola-Malpighi Polyclinic, Department of Medical and Surgical Sciences (DIMEC), University of Bologna, Via Massarenti 9, 40138 Bologna, BO, Italy.

[6]CNR Researcher, Institute of Biomembranes, Bioenergetics and Molecular Biotechnologies (IBIOM)
Via Amendola 165/A, I-70126, Bari, Italy.

[7]Department of Medical and Biological Sciences, University of Udine, P.le Massimiliano Kolbe 4, 33100 Udine, Italy.

[8]Department of Chemistry, University of Florence, Via della Lastruccia 3, 50019 Sesto Fiorentino, Florence, Italy.

*Corresponding Author

E-mail: pierluigi.strippoli@unibo.it

Phone: +39 0512094117

Fax: +39 0512094110

A

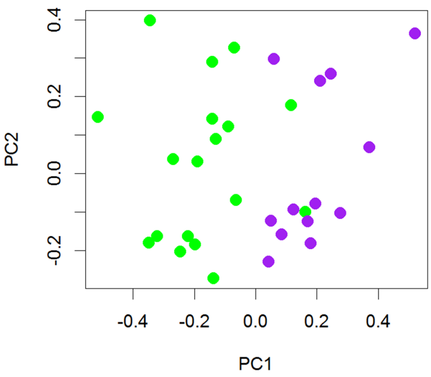

Discrimination accuracy: 73.6%

Confusion matrix:

	DS	CTRL
DS	**0.83**	0.17
CTRL	0.39	**0.61**

B

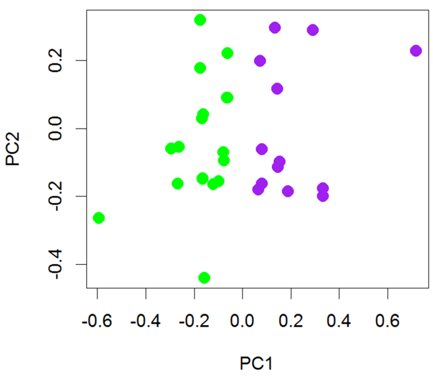

Discrimination accuracy: 77.2%

Confusion matrix:

	DS	CTRL
DS	**0.79**	0.21
CTRL	0.26	**0.74**

C

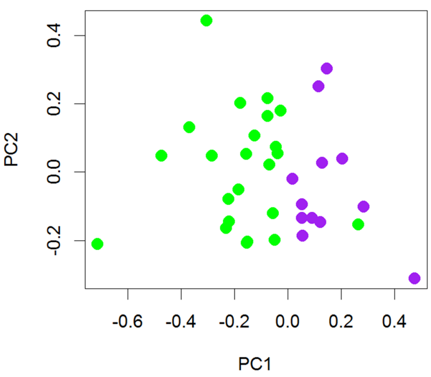

Discrimination accuracy: 75.4%

Confusion matrix:

	DS	CTRL
DS	**0.85**	0.15
CTRL	0.44	**0.56**

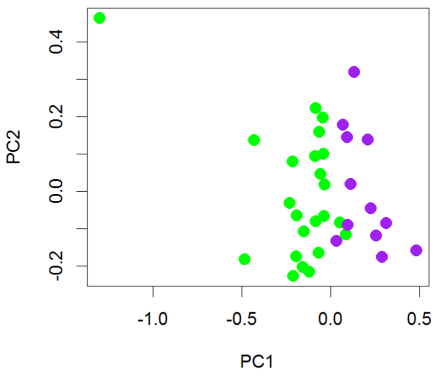

Discrimination accuracy: 84.7%

Confusion matrix:

	DS	CTRL
DS	**0.91**	0.09
CTRL	0.27	**0.73**

Supplementary Figure S1. **PLS-CA analysis of plasma samples by sex**. Score plot, PC1 vs. PC2, and corresponding confusion matrices. In the score plot, each dot represents a different plasma sample (all samples). Green dots: DS samples; purple dots: healthy controls. **(A)** and **(B)**: females (DS, n=18; CTRL, n=13). **(A)** 1Dpresat CPMG spectra**;** discrimination accuracy: 73.6% (95% CI, 72.9-74.3%); sensitivity: 82.7% (95% CI, 81.8-83.5%); specificity: 61.1% (95% CI, 59.9-62.3%). **(B)** 1Dpresat NOESY spectra; discrimination accuracy: 77.2% (95% CI, 76.5-77.9%); sensitivity: 79.2% (95% CI, 78.3-80.2%); specificity: 74.5% (95% CI, 73.5-75.5%). **(C)** and **(D)**: males (DS, n=23; CTRL, n=12). **(C)** 1Dpresat CPMG spectra; discrimination accuracy: 75.4% (95% CI, 74.7-76.1%); sensitivity: 85.4% (95% CI, 84.6-86.2%); specificity: 56.3% (95% CI, 54.9-57.6%). **(D)** 1Dpresat NOESY spectra; discrimination accuracy: 84.7% (95% CI, 84.1-85.32%); sensitivity: 90.9% (95% CI, 90.3-91.5%); specificity: 72.7% (95% CI, 71.4-74.0%).

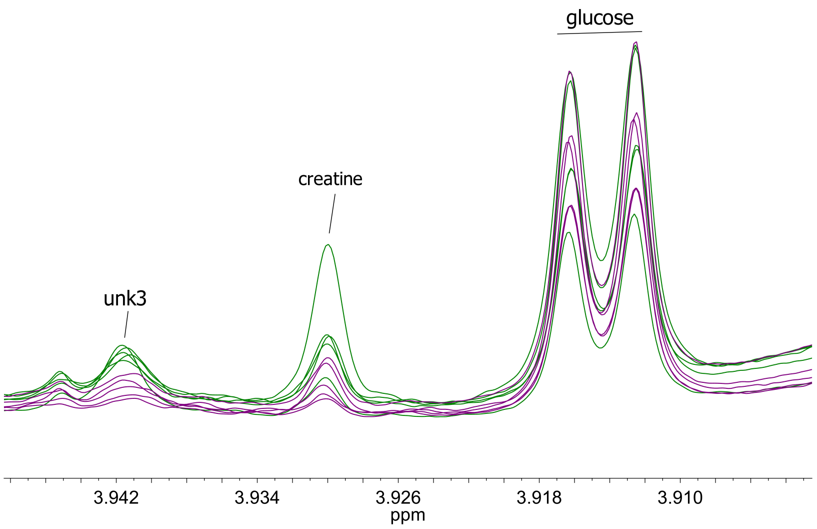

Supplementary Figure S2. Unk3 (unknown metabolite) NMR resonance in ^{1}H CPMG spectra. NMR spectra from 5 DS patients (green traces) and 5 healthy control samples (purple traces), taken as example.

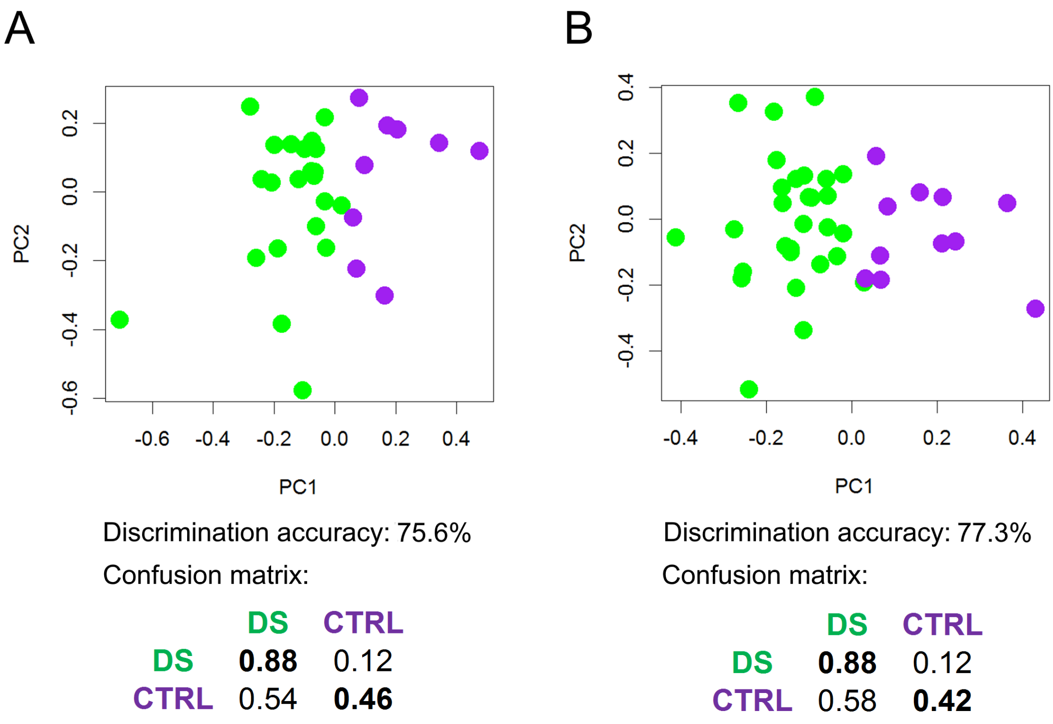

Supplementary Figure S3. PLS-CA analysis of urine samples by sex. Score plot, PC1 vs. PC2, and corresponding confusion matrices. In the score plot, each dot represents a different urine sample. Green dots: DS samples; purple dots: healthy controls. **(A)** Females (DS, n=22; CTRL, n=9); discrimination accuracy: 75.6% (95% CI, 74.9-76.2%); sensitivity: 87.8% (95% CI, 87.1-88.4%); specificity: 45.7% (95% CI, 44.1-47.3%)**.** **(B)** Males (DS, n=29; CTRL, n=11); discrimination accuracy: 77.3% (95% CI, 76.6-77.9%); sensitivity: 87.6% (95% CI, 86.9-88.3%); specificity: 42.4% (95% CI, 40.6-44.1%).

Supplementary Table S1. List of identified metabolites in plasma samples with respective ^{1}H chemical shift values. The compound IDs derived from the Human Metabolome Database (HMDB) are provided. The multiplicity of each signal is also reported; s: singlet; d: doublet; t: triplet; dd: doublet of doublets; m: multiplet.

Metabolite	HMBD	δ (^{1}H chemical shifts) ppm
2-Hydroxybutyrate	HMDB0000008	3.99 (dd); 1.73 (m);1.64 (m);0.89 (t)
3-Hydroxybutyrate	HMDB0000357	4.16 (m); 2.41 (m); 2.31 (m); 1.19 (d)
Acetate	HMDB0000042	1.91 (s)
Acetoacetate	HMDB0000060	3.44 (s); 2.27 (s)
Acetone	HMDB0001659	2.22 (s)
L-Alanine	HMDB0000161	3.76 (q); 1.46 (d)
Citrate	HMDB0000094	2.65 (d); 2.53 (d)
Creatine	HMDB0000064	3.92 (s); 3.02 (s)
Creatinine	HMDB0000562	4.05 (s); 3.03 (s)
Formate	HMDB0000142	8.44 (s)
Fumarate	HMDB0000134	6.51 (s)
D-Glucose	HMDB0000122	5.24 (d); 4.63 (d); 3.89 (dd); 3.82 (m); 3.72 (m); 3.52 (dd); 3.46 (m); 3.40 (m); 3.23 (dd)
L-Glutamate	HMDB0000148	3.75 (dd); 2.34 (m); 2.12 (m); 2.04 (m)
L-Glutamine	HMDB0000641	3.77 (t); 2.45 (m); 2.12 (m)
Glycerol	HMDB0000131	3.77 (m); 3.64 (m); 3.55 (m)
L-Glycine	HMDB0000123	3.56 (s)
L-Histidine	HMDB0000177	7.89 (d); 7.08 (d); 3.98 (dd); 3.23 (dd); 3.16 (dd)
L-Isoleucine	HMDB0000172	3.66 (d); 1.97 (m); 1.46 (m); 1.25 (m); 1.00 (d); 0.93 (t)
Lactate	HMDB0000190	4.10 (q); 1.32 (d)
L-Leucine	HMDB0000687	3.72 (m); 1.70 (m); 0.95 (t)
L-Lysine	HMDB0000182	3.74 (m); 3.02 (m); 1.89 (m); 1.71 (m); 1.46 (m)
D-Mannose	HMDB0000169	5.17 (d); 4.89 (d); 3.92 (m); 3.88 (dd); 3.84 (m); 3.80 (m); 3.74 (m); 3.65 (m); 3.56 (t); 3.37 (m)
L-Methionine	HMDB0000696	3.85 (dd); 2.63 (t); 2.16 (m)
L-Phenylalanine	HMDB0000159	7.42 (m); 7.36 (m); 7.32 (d); 3.98 (dd); 3.19 (m)
Pyruvate	HMDB0000243	2.36 (s)
Succinate	HMDB0000254	2.39 (s)
L-Threonine	HMDB0000167	4.25 (m); 3.58 (d); 1.32 (d)
L-Tyrosine	HMDB0000158	7.17 (m); 6.88 (m); 3.92 (dd); 3.17 (dd); 3.02 (dd)
Unk1	-	1.13 (d)
Unk2	-	1.43 (d)
Unk3	-	3.94 (s)
L-Valine	HMDB0000883	3.60 (d); 2.26 (m); 1.03 (d); 0.98 (d)

Supplementary Table S2. Univariate and multivariate logistic regression. ODD ratios for the comparison between plasma samples from DS and control subjects are reported. For the calculation of the multivariate ODD ratios and respective p-values, each metabolite was considered together with age and sex variables in the analysis.

**Metabolite**	**ODD ratio*** *univariate	**p-value*** *univariate	**ODD ratio**** **Adjusted for age and sex	**p-value**** **Adjusted for age and sex
Pyruvate	0.22	0.007	0.26	0.029
Glutamine	0.27	0.018	0.27	0.027
Creatine	0.16	0.001	0.27	0.043
Formate	0.22	0.007	0.26	0.022
Unk3	0.16	0.002	0.20	0.006
Succinate	0.16	0.002	0.19	0.006
Acetone	0.33	0.039	0.29	0.037
Tyrosine	2.51	0.079	3.33	0.045
Glycerol	0.16	0.002	0.15	0.004
Acetate	0.33	0.039	0.29	0.037

Supplementary Table S3. List of identified metabolites in urine samples with respective ^{1}H chemical shift values. The compound IDs derived from the Human Metabolome Database (HMDB) are provided. The multiplicity of each signal is also reported; s: singlet; d: doublet; t: triplet; dd: doublet of doublets; m: multiplet.

Metabolite	HMBD	δ (^{1}H chemical shifts) ppm
1-Methylnicotinamide	HMDB0000699	9.28 (s); 8.96 (d); 8.89 (d); 8.18 (t); 4.47 (s)
2-Hydroxybutyrate	HMDB0000008	3.99 (dd); 1.73 (m);1.64 (m);0.89 (t)
3-Hydroxyisovalericacid	HMDB0000754	2.35 (s); 1.26 (s)
4-Hydroxyphenylacetate	HMDB0000020	7.15 (d); 6.85 (d); 3.44 (s)
Acetone	HMDB0001659	2.22 (s)
L-Alanine	HMDB0000161	3.76 (q); 1.46 (d)
Allantoin	HMDB0000462	5.38 (s)
L-Asparagine	HMDB0000168	4.00 (dd); 2.94 (m); 2.84 (m)
Citrate	HMDB0000094	2.65 (d); 2.53 (d)
Creatine	HMDB0000064	3.92 (s); 3.02 (s)
Creatinine	HMDB0000562	4.05 (s); 3.03 (s)
Dimethylamine	HMDB0000087	2.70 (s)
Dimethylglycine	HMDB0000092	3.71 (s); 2.91 (s)
Ethanolamine	HMDB0000149	3.81 (t); 3.13 (t)
Formate	HMDB0000142	8.44 (s)
Fumarate	HMDB0000134	6.51 (s)
L-Glutamate	HMDB0000148	3.75 (dd); 2.34 (m); 2.12 (m); 2.04 (m)
L-Glutamine	HMDB0000641	3.77 (t); 2.45 (m); 2.12 (m)
L-Glycine	HMDB0000123	3.56 (s)
Glycolate	HMDB0000115	3.94 (s)
Hippurate	HMDB0000714	7.83 (m); 7.62 (m); 7.54 (m); 3.96 (d)
L-Isoleucine	HMDB0000172	3.66 (d); 1.97 (m); 1.46 (m); 1.25 (m); 1.00 (d); 0.93 (t)
L-Leucine	HMDB0000687	3.72 (m); 1.70 (m); 0.95 (t)
L-Lysine	HMDB0000182	3.74 (m); 3.02 (m); 1.89 (m); 1.71 (m); 1.46 (m)
Phenylacetylglycine	HMDB0000821	7.41 (m); 7.37 (m); 7.35 (m); 3.74 (d); 3.67 (s)
Taurine	HMDB0000251	3.25 (t); 3.42 (t)
Trigonelline	HMDB0000875	9.11 (s); 8.82 (m);8.01 (m); 4.43 (s)
Trimethylamine-N-Oxide	HMDB0000925	3.24 (s)
L-Tyrosine	HMDB0000158	7.17 (m); 6.88 (m); 3.92 (dd); 3.17 (dd); 3.02 (dd)
L-Valine	HMDB0000883	3.60 (d); 2.26 (m); 1.03 (d); 0.98 (d)

Supplementary Table S4. Genomic location of genes encoding for the enzymes upstream or downstream of the metabolites with altered concentration levels in DS plasma or urine.

**Metabolite / KeGG Entry**	**Metabolic Pathways**	**Enzyme EC number (group)**	**Enzyme (Official Full Gene Name)**	**Gene Symbol**	**Location**
Acetate C00033	Glycolysis/ Gluconeogenesis	1.2.1.3 (Aldehyde-DH NAD+)	Aldehyde dehydrogenase 2 family (mitochondrial)	*ALDH2*	12q24.12
			Aldehyde dehydrogenase 1 family member B1	*ALDH1B1*	9p13.1
			Aldehyde dehydrogenase 9 family member A1	*ALDH9A1*	1q24.1
			Aldehyde dehydrogenase 3 family member A2	*ALDH3A2*	17p11.2
			Aldehyde dehydrogenase 7 family member A1	*ALDH7A1*	5q23.2
	Glycolysis/ Gluconeogenesis	1.2.1.5 (Aldehyde-DH NAD(P)+)	Aldehyde dehydrogenase 3 family member A1	*ALDH3A1*	17p11.2
			Aldehyde dehydrogenase 1 family member A3	*ALDH1A3*	15q26.3
			Aldehyde dehydrogenase 3 family member B1	*ALDH3B1*	11q13.2
			Aldehyde dehydrogenase 3 family member B2	*ALDH3B2*	11q13.2
	Glycolysis/ Gluconeogenesis	6.2.1.1 (Acetyl-CoA Syntase)	Acyl-CoA synthetase short chain family member 2	*ACSS2*	20q11.22
			Acyl-CoA synthetase short chain family member 1	*ACSS1*	20p11.21
	Pyruvate metabolism	3.1.2.1	Acyl-CoA thioesterase 12	*ACOT12*	5q14.1
	Pyruvate metabolism	3.6.1.7 (Acylphospatase)	Acylphosphatase 1	*ACYP1*	14q24.3
			Acylphosphatase 2	*ACYP2*	2p16.2
Acetoacetate C00164	Tyrosine metabolism	3.7.1.2	Fumarylacetoacetate hydrolase	*FAH*	15q25.1
	Synthesis and degradation of ketone bodies	1.1.1.30 (3-Hydroxybutyrate DH)	3-hydroxybutyrate dehydrogenase 2	*BDH2*	4q24
	Butanoate metabolism		3-hydroxybutyrate dehydrogenase 1	*BDH1*	3q29
	Synthesis and degradation of ketone bodies	4.1.3.4 (Hydroxy-3-methylglutaryl-CoA Lyase)	3-hydroxymethyl-3-methylglutaryl-CoA lyase	*HMGCL*	1p36.11
	Valine, leucine and isoleucine degradation				
	Butanoate metabolism		3-hydroxymethyl-3-methylglutaryl-CoA lyase like 1	*HMGCLL 1*	6p12.1
	Synthesis and degradation of ketone bodies	2.8.3.5 (3-Oxoacid CoA-Transferase)	3-oxoacid CoA-transferase 1	*OXCT1*	5p13.1
	Valine, leucine and isoleucine degradation				
	Butanoate metabolism		3-oxoacid CoA-transferase 2	*OXCT2*	1p34.2

	Valine, leucine and isoleucine degradation	6.2.1.16	Acetoacetyl-CoA synthetase	*AACS*	12q24.31
	Butanoate metabolism				
Citrate C00158	Citrate cycle (TCA cycle)	2.3.3.1	citrate synthase	*CS*	12q13.3
	Glyoxylate and dicaboxylate metabolism				
	Citrate cycle (TCA cycle)	2.3.3.8	ATP citrate lyase	*ACLY*	17q21.2
	Citrate cycle (TCA cycle)	4.2.1.3 (Aconitase)	Aconitase 1	*ACO1*	9p21.1
	Glyoxylate and dicaboxylate metabolism		Aconitase 2	*ACO2*	22q13.2
	Alanine, aspartate and glutamate metabolism	6.3.1.17	Ribosomal modification protein rimK like family member B	*RIMKLB*	12p13.31
Creatine C00300	Glycine, serine and threonine metabolism	2.1.1.2	uanidinoacetate N-methyltransferase	*GAMT*	19p13.3
	Arginine and proline metabolism				
	Arginine and proline metabolism	2.7.3.2 (Creatine Kinase)	Creatine kinase B	*CKB*	14q32.33
			Creatine kinase, M-type	*CKM*	19q13.32
			Creatine kinase, mitochondrial 1B	*CKMT1B*	15q15.3
			Creatine kinase, mitochondrial 2	*CKMT2*	5q14.1
			Creatine kinase, mitochondrial 1A	*CKMT1A*	15q15.3
Ethanolamin e C00189	Glycerophospholip id metabolism	3.1.3.75	Phosphoethanolamine/Phosphoc oline phosphatase	*PHOSPH O1*	17q21.32
	Glycerophospholip id metabolism	2.7.1.82 (Ethanolamine kinase)	Choline kinase alpha	*CHKA*	11q13.2
			Choline kinase beta	*CHKB*	22q13.33
			Ethanolamine kinase 2	*ETNK2*	1q32.1
			Ethanolamine kinase 1	*ETNK1*	12p12.1
Formate C00058	Glyoxylate and dicarboxylate metabolism	3.5.1.9	Arylformamidase	*AFMID*	17q25.5
Fumarate C00122	Citrate cycle (TCA cycle)	4.2.1.2	Fumarate hydratase	*FH*	1q43
	Pyruvate metabolism				
	Citrate cycle (TCA cycle)	1.3.5.1 (Succinate dehydrogenase complex flavoprotein subunit A)	Succinate dehydrogenase complex flavoprotein subunit A	*SDHA*	5p15.33
			Succinate dehydrogenase complex iron sulfur subunit B	*SDHB*	1p36.13
	Oxydative phosphorylation		Succinate dehydrogenase complex subunit C	*SDHC*	1q23.3
			Succinate dehydrogenase complex subunit D	*SDHD*	11q23.1

	Arginine biosynthesis	4.3.2.1	Argininosuccinate lyase	*ASL*	7q11.21
	Alanine, aspartate and glutamate metabolism				
	Alanine, aspartate and glutamate metabolism	4.3.2.2	Adenylosuccinate lyase	*ADSL*	22q13.1
	Tyrosine metabolism	3.7.1.2	Fumarylacetoacetate hydrolase	*FAH*	15q25.1
	Tyrosine metabolism	3.7.1.5	Fumarylacetoacetate hydrolase domain containing 1	*FAHD1*	16p13.3
Glycerol C00116	Galactose metabolism	3.2.1.22	Galactosidase alpha	*GLA*	Xq22.1
	Glycerolipid metabolism	1.1.1.2	Aldo-keto reductase family 1 member A1	*AKR1A1*	1p34.1
	Glycerolipid metabolism	1.1.1.21 (Aldehyde Reductase)	Aldo-keto reductase family 1 member B	*AKR1B1*	7q33
			Aldo-keto reductase family 1 member B10	*AKR1B10*	7q33
	Glycerolipid metabolism	2.7.1.30 (Glycerol Kinase)	Glycerol kinase	*GK*	Xp21.2
			Glycerol kinase 2	*GK2*	4q21.21
	Glycerolipid metabolism	3.1.1.23	Acylglycerol lipase	*MGLL*	3q21.3
Glycine C00037	Primary bile acid biosynthesis	2.3.1.65	Bile acid-CoA:amino acid N-acyltransferase	*BAAT*	9q31.1
	Glyoxylate and dicarboxilate metabolism	1.8.1.4	Dihydrolipoamide dehydrogenase	*DLD*	7q31.1
	Aminoacyl-tRNA biosynthesis	6.1.1.14	Glycyl-tRNA synthetase	*GARS*	7p14.3
	Glutathione metabolism	3.4.11.2	Alanyl aminopeptidase, membrane	*ANPEP*	15q26.1
	Glutathione metabolism	3.4.11.1<br>3.4.11.5	Leucine aminopeptidase 3	*LAP3*	4p15.32
	Glutathione metabolism	6.3.2.3	Glutathione synthetase	*GSS*	20q11.22
	Glycine, serine and threonine metabolism	2.6.1.44<br>2.6.1.45<br>(Alanine-glyoxylate aminotransaminase)	Alanine-glyoxylate aminotransferase	*AGXT*	2q37.3
	Glyoxylate and dicarboxilate metabolism		Alanine-glyoxylate aminotransferase 2	*AGXT2*	5p13.2
	Glycine, serine and threonine metabolism	2.1.2.1<br>(serine hydroxymethyltransfer ase)	Serine hydroxymethyltransferase 1	*SHMT1*	17p11.2
	Glyoxylate and dicarboxilate metabolism		Serine hydroxymethyltransferase 2	*SHMT2*	12q13.3
	Glycine, serine and threonine metabolism	1.5.3.1	Pipecolic acid and sarcosine oxidase	*PIPOX*	17q11.2
	Glycine, serine and threonine metabolism	1.5.8.3	Sarcosine dehydrogenase	*SARDH*	9q34.2
	Glycine, serine and threonine metabolism	2.1.1.20	Glycine N-methyltransferase	*GNMT*	6p21.1

	Glycine, serine and threonine metabolism	2.3.1.29	Glycine C-acetyltransferase	*GCAT*	22q13.1
	Glycine, serine and threonine metabolism	1.4.3.3	D-amino acid oxidase	*DAO*	12q24.11
	Glycine, serine and threonine metabolism	1.4.4.2	Glycine decarboxylase	*GLDC*	9p24.1
	Glycine, serine and threonine metabolism	2.3.1.37 (5'-aminolevulinate synthase)	5'-aminolevulinate synthase 1	*ALAS1*	3p21.2
	Porphyrin and chlorophyll metabolism		5'-aminolevulinate synthase 2	*ALAS2*	Xp11.21
	Glycine, serine and threonine metabolism	2.1.4.1	Glycine amidinotransferase	*GATM*	15q21.1
Hippurate C01586	Phenylalanine metabolism	2.3.1.71	Glycine-N-acyltransferase	*GLYAT*	11q12.1
Lactate C00186	Glycolysis/ Gluconeogenesis	1.1.1.27 (Lactate dehydrogenase)	Lactate dehydrogenase A like 6A	*LDHAL6A*	11p15.1
			Lactate dehydrogenase A	*LDHA*	11p15.1
	Pyruvate metabolism		Lactate dehydrogenase B	*LDHB*	12p12.1
			Lactate dehydrogenase C	*LDHC*	11p15.1
			Lactate dehydrogenase A like 6B	*LDHAL6B*	15q22.2
L-Glutamic acid C00025	Glutathione metabolism	2.3.2.2 3.4.19.13 (gamma-glutamyltransferase)	Gamma-glutamyltransferase 6	*GGT6*	17p13.2
			Gamma-glutamyltransferase 1	*GGT1*	22q11.23
			Gamma-glutamyltransferase 7	*GGT7*	20q11.22
			Gamma-glutamyltransferase 5	*GGT5*	22q11.23
	Alanine, aspartate and glutamate metabolism	3.5.1.2 (glutaminase)	Glutaminase	*GLS*	2q32.2
	D-glutamine and D-glutamate metabolism		Glutaminase 2	*GLS2*	12q13.3
	Glutathione metabolism	6.3.3.2 6.3.2.2 (glutamate-cysteine ligase subunit)	Glutamate-cysteine ligase catalytic subunit	*GCLC*	6p12.1
			Glutamate-cysteine ligase modifier subunit	*GCLM*	1p22.1
	Glutathione metabolism	3.5.2.9	5-oxoprolinase (ATP-hydrolysing)	*OPLAH*	8q24.3
	Arginine biosynthesis	1.4.1.3 (Glutamate DH NAD (P)+)00	Glutamate dehydrogenase 1	*GLUD1*	10q23.2
	Alanine, aspartate and glutamate metabolism				
	D-glutamine and D-glutamate metabolism		Glutamate dehydrogenase 2	*GLUD2*	Xq24

	Nitrogen metabolism				
	Arginine biosynthesis	2.6.1.1 (glutamic-oxaloacetic transaminase)	Glutamic-oxaloacetic transaminase 1	*GOT1*	10q24.2
			Glutamic-oxaloacetic transaminase 2	*GOT2*	16q21
	Arginine biosynthesis	2.6.1.2 (glutamic-pyruvic transaminase)	Glutamic-pyruvic transaminase	*GPT*	8q24.3
			Glutamic-pyruvic transaminase 2	*GPT2*	16q11.2
	Arginine biosynthesis	2.3.1.1	N-acetylglutamate synthase	*NAGS*	17q21.31
	Glycoxylate and dicarboxylate metabolism	6.3.1.2	Glutamate-ammonia ligase	*GLUL*	1q25.3
	Alanine, aspartate and glutamate metabolism				
	Alanine, aspartate and glutamate metabolism	1.2.1.88	Aldehyde dehydrogenase 4 family member A1	*ALDH4A1*	1p36.13
	Arginine and proline metabolism				
	Alanine, aspartate and glutamate metabolism	4.1.1.15 (Glutamate decarboxylase)	Glutamate decarboxylase 1	*GAD1*	2q31.1
	Butanoate metabolism		Glutamate decarboxylase 2	*GAD2*	10p12.1
	Arginine and proline metabolism	2.7.2.11	Aldehyde dehydrogenase 18 family member A1	*ALDH18A1*	10q24.1
	Histidine metabolism	2.1.2.5	Formimidoyltransferase cyclodeaminase	*FTCD*	21q22.3
	Porphyrin and chlorophyll metabolism	6.1.1.17	Glutamyl-tRNA synthetase	*EARS2*	16p12.2
	Aminoacyl-tRNA biosynthesis		Bifunctional glutamyl/prolyl-tRNA synthetase	*EPRS*	1q41
L-Glutamine C00064	Arginine biosynthesis	3.5.1.2 (Glutaminase)	Glutaminase	*GLS*	2q32.2
	Alanine, aspartate and glutamate metabolism				
	D-glutamine and D-glutamate metabolism		Glutaminase 2	*GLS2*	12q13.3
	Arginine biosynthesis	6.3.1.2	Glutamate-ammonia ligase	*GLUL*	1q25.3
	Alanine, aspartate and glutamate metabolism				
	Glycoxylate and dicarboxylate metabolism				

	Nitrogen metabolism				
	Alanine, aspartate and glutamate metabolism	2.6.1.16 (Glucosamine-Fructose 6P-Amino-Transferase)	Glutamine-fructose-6-phosphate transaminase 1	*GFPT1*	2p13.3
			Glutamine-fructose-6-phosphate transaminase 2	*GFPT2*	5q35.3
	Purine metabolism	2.4.2.14	Phosphoribosyl pyrophosphate amidotransferase	*PPAT*	4q12
	Alanine, aspartate and glutamate metabolism				
	Aminoacyl-tRNA biosynthesis	6.1.1.18	Glutaminyl-tRNA synthetase	*QARS*	3p21.31
	Alanine, aspartate and glutamate metabolism	6.3.5.5	Carbamoyl-phosphate synthetase 2, aspartate Transcarbamylase, and dihydroorotase	*CAD*	2p23.3
L-Isoleucine C00407	Valine, leucine and isoleucine degradation	1.4.3.1	Interleukin 4 induced 1	*IL4I1*	19q13.33
	Valine, leucine and isoleucine degradation	2.6.1.42 (Branched chain amino acid transaminase)	Branched chain amino acid transaminase 1	*BCAT1*	12p12.1
	Valine, leucine and isoleucine biosynthesis		Branched chain amino acid transaminase 2	*BCAT2*	19q13.33
	Aminoacyl-tRNA biosynthesis	6.1.1.5 (Isoleucyl-tRNA synthetase)	Isoleucyl-tRNA synthetase	*IARS*	9q22.31
			Isoleucyl-tRNA synthetase 2, mitochondrial	*IARS2*	1q41
L-Leucine C00123	Valine, leucine and isoleucine degradation	2.6.1.42 (Branched-chain Aminoacid-Amino-Transferase)	Branched chain amino acid transaminase 1	*BCAT1*	12p12.1
	Valine, leucine and isoleucine biosynthesis		Branched chain amino acid transaminase 2	*BCAT2*	19q13.33
	Aminoacyl-tRNA biosynthesis	6.1.1.4	Leucyl-tRNA synthetase 2, mitochondrial	*LARS2*	3p21.31
L-Lysine C00047	Aminoacyl-tRNA biosynthesis	6.1.1.6	Lysyl-tRNA synthetase	*KARS*	16q23.1
	Biotin metabolism	3.5.1.12	Biotinidase	*BTD*	3p25.1
	Lysine degradation	1.5.1.8	Alpha-aminoadipic semialdehyde synthase	*AASS*	7q31.32
Phenylacetyl-glycine C05598	Phenylalanine metabolism	2.3.1.13	Glycine-N-acyltransferase	*GLYAT*	11q12.1
Pyruvate C00022	Cysteine and methionine metabolism	2.8.1.2 (sulfurtransferase)	Mercaptopyruvate sulfurtransferase	*MPST*	22q12.3
			Thiosulfate sulfurtransferase	*TST*	22q12.3
	Alanine, aspartate and glutamate metabolism	2.6.1.2 (glutamic-pyruvic transaminase)	Glutamic-pyruvic transaminase	*GPT*	8q24.3
			Glutamic-pyruvic transaminase 2	*GPT2*	16q11.2
	Alanine, aspartate and glutamate metabolism	2.6.1.44 (alanine-glyoxylate aminotransferase)	Alanine-glyoxylate aminotransferase	*AGXT*	2q37.3
			Alanine-glyoxylate aminotransferase 2	*AGXT2*	5p13.2
	Cysteine and	4.4.1.1	Cystathionine gamma-lyase	*CTH*	1p31.1

	methionine metabolism				
	Glycine, serine and threonine metabolism	4.3.1.17<br>4.3.1.19<br>(serine dehydratase)	Serine dehydratase	*SDS*	12q24.13
			Serine dehydratase like	*SDSL*	12q24.13
	Arginine and proline metabolism<br>Glycoxylate and dicarboxylate metabolism	4.1.3.16	4-hydroxy-2-oxoglutarate aldolase 1	*HOGA1*	10q24.2
	Tyrosine metabolism	3.7.1.5	Fumarylacetoacetate hydrolase domain containing 1	*FAHD1*	16p13.3
	Glycolysis / Gluconeogenesis	1.1.1.27<br>(L-Lactate DH)	Lactate dehydrogenase A like 6A	*LDH6A*	11p15.1
			Lactate dehydrogenase A	*LDHA*	11p15.1
	Pyruvate metabolism		Lactate dehydrogenase B	*LDHB*	12p12.1
			Lactate dehydrogenase C	*LDHC*	11p15.1
			Lactate dehydrogenase A like 6B	*LDH6B*	15q22.2
	Glycolysis / Gluconeogenesis	2.7.1.40<br>(Pyruvate Kinase)	Pyruvate kinase, liver and RBC	*PKLR*	1q22
	Pyruvate metabolism		Pyruvate kinase, muscle	*PKM*	15q23
	Glycolysis / Gluconeogenesis	1.2.4.1<br>(Pyruvate DH)	Pyruvate dehydrogenase alpha 1	*PDHA1*	Xp22.12
	Citrate cycle (TCA cycle)		Pyruvate dehydrogenase alpha 2	*PDHA2*	4q22.3
	Pyruvate metabolism		Pyruvate dehydrogenase (lipoamide) beta	*PDHB*	3p14.3
	Pyruvate metabolism	1.1.2.4	Lactate dehydrogenase D	*LDHD*	16q23.1
	Citrate cycle (TCA cycle)<br>Pyruvate metabolism	6.4.1.1	Pyruvate Carboxylase	*PC*	11q13.2
	Pyruvate metabolism	1.1.1.38<br>(malic enzyme)	Malic enzyme 2	*ME2*	18q21.2
	Pyruvate metabolism	1.1.1.40<br>(malic enzyme)	Malic enzyme 3	*ME3*	11q14.2
			Malic enzyme 1	*ME1*	6q14.2
Succinate C00042	Alanine, aspartate and glutamate metabolism<br>Butanoate metabolism	1.2.1.24	Aldehyde dehydrogenase 5 family member A1	*ALDH5A1*	6p22.3
	Citrate cycle (TCA cycle)	1.3.5.1<br>(Succinate DH (ubiquinone)	Succinate dehydrogenase complex flavoprotein subunit A	*SDHA*	5p15.33
			Succinate dehydrogenase complex iron sulfur subunit B	*SDHB*	1p36.13
	Oxidative phosphorilation		Succinate dehydrogenase complex subunit C	*SDHC*	1q23.3
			Succinate dehydrogenase complex subunit D	*SDHD*	11q23.1
	Citrate cycle (TCA cycle)	6.2.1.4	Succinate-CoA ligase GDP-forming beta subunit	*SUCLG2*	3p14.1

	Propanoate metabolism				
	Citrate cycle (TCA cycle)	6.2.1.5 (Succinil-CoA Syntase α-β subunits)	Succinate-CoA ligase alpha subunit	*SUCLG1*	2p11.2
	Propanoate metabolism		Succinate-CoA ligase ADP-forming beta subunit	*SUCLA2*	13q14.2
Threonine C00188	Glycine, serine and threonine metabolism	4.3.1.19 (Serine dehydratase)	Serine dehydratase	*SDS*	12q24.13
	Valine, leucine and isoleucine biosynthesis		Serine dehydratase like	*SDSL*	12q24.13
	Aminoacyl-tRNA biosynthesis	6.1.1.3 (Threonyl-tRNA synthetase)	Threonyl-tRNA synthetase like 2	*TARSL2*	15q26.3
			Threonyl-tRNA synthetase	*TARS*	5p13.3
			Threonyl-tRNA synthetase 2, mitochondrial	*TARS2*	1q21.2
Tyrosine C00082	Aminoacyl-tRNA biosynthesis	6.1.1.1 (tyrosyl-tRNA synthetase)	Tyrosyl-tRNA synthetase	*YARS*	1p35.1
			Tyrosyl-tRNA synthetase 2	*YARS2*	12p11.21
	Tyrosine metabolism	2.6.1.1 (glutamic-oxaloacetic transaminase)	Glutamic-oxaloacetic transaminase 1	*GOT1*	10q24.2
	Phenylalanine, tyrosine and tryptophan biosynthesis		Glutamic-oxaloacetic transaminase 2	*GOT2*	16q21
	Ubiquinone and other terpenoid-quinone biosinthesis	2.6.1.5	Tyrosine aminotransferase	*TAT*	16q22.2
	Tyrosine metabolism				
	Phenylalanine, tyrosine and tryptophan biosynthesis				
	Tyrosine metabolism	1.4.3.2	Interleukin 4 induced 1	*IL4I1*	19q13.33
	Phenylalanine, tyrosine and tryptophan biosynthesis				
	Tyrosine metabolism	1.14.18.1	Tyrosinase	*TYR*	11q14.3
	Tyrosine metabolism	1.14.16.2	Tyrosine hydroxylase	*TH*	11p15.5
	Tyrosine metabolism	4.1.1.28	Dopa decarboxylase	*DDC*	7p12.2-p12.1
	Tyrosine metabolism	1.11.1.8	Thyroid peroxida	*TPO*	2p25.3
	Phenylalanine metabolism	1.14.16.1	Phenylalanine hydroxylase	*PAH*	12q23.2
	Phenylalanine, tyrosine and tryptophan biosynthesis				
